# Knock-down of the long isoform of the WNK1 kinase mitigates the anti-glomerular basement membrane glomerulonephritis in mice

**DOI:** 10.1038/s41598-026-36715-8

**Published:** 2026-02-05

**Authors:** Cyril Mousseaux, Tiffany Migeon, Perrine Frère, Nadhir Yousfi, Souhila Ouchelouche, Thomas Mouche, Marie-Christine Verpont, Brigitte Surin, Liliane Louedec, David Buob, Pierre Galichon, Juliette Hadchouel

**Affiliations:** 1 Institut National de la Santé et de la Recherche Médicale (Inserm), Sorbonne Université, Common and Rare Kidney Diseases: from Molecular Events to Precision Medicine, CoRaKiD, 75020 Paris, France; 2 Soins Intensifs Néphrologiques et Rein Aigu, Tenon Hospital, Assistance Publique, Hôpitaux de Paris, Paris, France; 3 Anatomie et Cytologie Pathologiques, Tenon Hospital, Assistance Publique, Hôpitaux de Paris, Paris, France; 4 Département de Transplantation et de Néphrologie, Pitié-Salpêtrière Hospital, Assistance Publique-Hôpitaux de Paris, Paris, France

**Keywords:** *WNK1*, Anti-GBM GN, RPGN, Podocyte, Glomerulus, Nephrology

## Abstract

The roles of the long isoform of the With No Lysine (K) 1 (L-WNK1) kinase have been mainly studied in the distal renal tubule, where it participates in sodium and chloride homeostasis. Yet, little is known about its role in its predominant expression site within the kidney, i.e. the glomerulus. We chose to study the consequences of L-WNK1 inhibition in a mouse model of rapidly progressive glomerulonephritis (RPGN), combining podocyte injury and parietal epithelial cell (PEC) activation. We show that L-WNK1 expression is upregulated in glomerular cells during RPGN in mice. A 50% reduction in L-WNK1 expression mitigates experimental nephrotoxic serum-induced RPGN in mice. Given that L-WNK1 is strongly expressed in podocytes, which play an important role in RPGN pathogenesis, we then studied a model of podocyte-specific knockdown of *WNK1*. Even if we could observe some changes in NTS-induced RPGN following this knock-down, the effects are not as marked as in the global inhibition model. These data suggest that L-WNK1 plays an important role in other glomerular cell types. Accordingly, we show in vitro that the inhibition of L-WNK1 activity impairs PECs migration. These results suggest that L-WNK1 could represent a new player in the pathophysiology of RPGN.

**Supplementary Information:**

The online version contains supplementary material available at 10.1038/s41598-026-36715-8.

## Introduction

With No Lysine (WNK) kinases are a family of serine/threonine kinases that includes four members (WNK1, WNK2, WNK3, and WNK4)^1^. In the kidney, the *WNK1* gene encodes two distinct proteins: a long isoform (L-WNK1) and a shorter, kinase-deficient isoform known as kidney-specific WNK1 (KS-WNK1)^2^. While KS-WNK1 is exclusively expressed in the distal nephron, L-WNK1 is expressed ubiquitously, throughout the body^3^. Large deletions in the first intron of the *WNK1* gene cause Familial Hyperkalemic Hypertension (FHHt), a rare mendelian form of hypertension. These mutations result in increased expression of L-WNK1 in the Distal Convoluted Tubule (DCT) and Connecting Tubule (CNT). Consequently, the activity of the Na^+^-Cl^−^ cotransporter (NCC) is elevated through a phosphorylation cascade downstream of L-WNK1, involving two kinases: oxidative stress-responsive kinase 1 (OSR1) and STE20-related proline/alanine-rich kinase (SPAK)^4,5^.

Although L-WNK1 has been predominantly studied in the DCT in the context of FHHt, its predominant physiological site of expression in the kidney is the glomerulus^6^. Transcriptomic analysis of mouse and human kidney revealed that L-WNK1 is highly expressed in podocytes, terminally differentiated epithelial cells that are crucial to the integrity of the glomerular filtration barrier^7,8^. A recent study highlighted the role of L-WNK1 in regulating the actin cytoskeleton in cultured podocytes and isolated mouse glomeruli^9^. The administration of a WNK inhibitor resulted in the alteration of the actin cytoskeleton, reduction of podocyte motility, and down-regulation of synaptopodin (an essential protein that interacts with actin)^10^.

Since no study has explored the role of L-WNK1 in the glomerulus in vivo, we aimed to investigate its functions in rapidly progressive glomerulonephritis (RPGN). RPGN is a life-threatening glomerular disease characterized by acute kidney injury, hematuria and proteinuria. Its pathogenesis involves the infiltration of inflammatory cells, necrotizing vasculitis, and podocyte injury, which triggers the activation of parietal epithelial cells (PECs)^11–13^. As a result, PECs proliferate and migrate within the glomerulus^14^, leading to the formation of glomerular crescents. Although the pathophysiology behind the formation of these crescents is not yet fully understood, the interaction between podocytes and PECs appears to play a crucial role. Supporting this hypothesis, even a single podocyte injury can trigger the activation and proliferation of PECs^13,15^.

Beyond its role in regulating sodium and chloride homeostasis, L-WNK1 is involved in several biological processes that could be involved in glomerulopathies. The acquisition of a proliferative phenotype is a hallmark of glomerular epithelial cells during RPGN. L-WNK1 has been shown to be essential for completing mitosis *in vitro via* its canonical partner OSR1^16^. Additionally, a recent study by Köchl and colleagues demonstrated that L-WNK1 is required for an immature subpopulation of thymocytes to progress through the G1 phase of the cell cycle^17^. Moreover, L-WNK1 has been reported to promote cell migration in various cell types, including T lymphocytes and corneal epithelial cells^18,19^.

Based on these data, we sought to determine the role of L-WNK1 in a murine model of RPGN. Our results show that L-WNK1 expression is elevated in the glomeruli of mice exposed to the anti-glomerular basement membrane glomerulonephritis (anti-GBM GN) model. We demonstrate that both global and podocyte-specific knock-down of L-WNK1 confer protection against glomerular injury. In cultured human podocytes, inhibition of L-WNK1 kinase activity reduces podocyte migration. Together, these findings reveal a novel and significant role for L-WNK1 in the kidney, beyond its known function in the distal nephron.

## Results

### L-WNK1 expression is increased during RPGN in mice

**Fig. 1:**
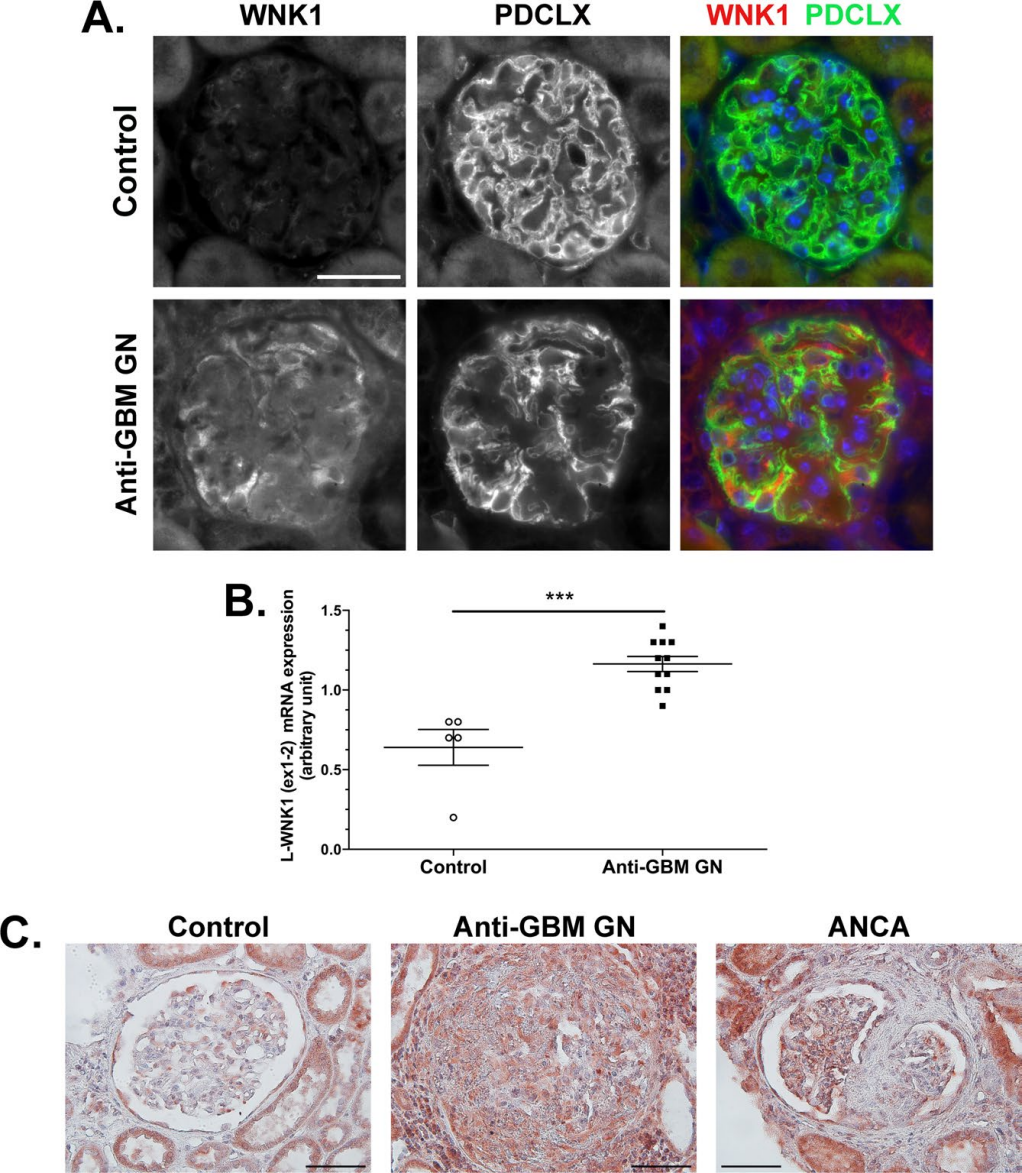
L-WNK1 is upregulated in glomerular cells following crescentic glomerulonephritis in mice. (**A**) Representative images showing immunofluorescent stainings of L-WNK1 (red) and podocalyxin (green) and DAPI (blue) at baseline and ten days after NTS injection (Anti-GBM GN)in wild-type mice. Scale bar: 25 μm. (**B**) Transcriptional expression of L-WNK1 is increased at day ten upon anti-GBM GN induction in wild-type mice(total kidney RNA). Data are presented as median +/- interquartile range (control: *n* = 5; Anti-GBM GN: *n* = 11). Mann-Whitney test, ***P < 0.001. (**C**) Representative images showing immunohistochemical staining of L-WNK1 (brown) from human kidneys: control kidney biopsy (Acute Tubular Necrosis), Anti-GBM GN (anti-glomerular basement membrane glomerulonephritis), ANCA vasculitis. Scale bar: 50 μm.

The anti-glomerular basement membrane glomerulonephritis (anti-GBM GN) model is widely used to mimic human RPGN in mice. Using an antibody recognizing both L-WNK1 and KS-WNK1, we observed in the control mouse kidney that L-WNK1 is expressed in the podocytes and PECs (Fig. 1A**)**. Induction of anti-GBM GN in wild-type mice showed increased level of L-WNK1 transcripts and protein in the glomerulus (Fig. 1A-B), ten days after the injection of NTS.

We then studied the expression of WNK1 in kidney biopsies from control and RPGN patients, i.e. patients with ANCA (Antineutrophil Cytoplasmic Autoantibody) vasculitis, and anti-glomerular basement membrane disease (Fig. 1C). In control human kidney biopsies, WNK1 is highly expressed in distal nephrons (reflecting the presence of KS-WNK1) but also in the cytoplasm of podocytes and PECs. In diseased kidneys, L-WNK1 expression is not only present in intraglomerular cells but also in inflammatory cells surrounding the glomeruli.

### *L-WNK1*^*+/−*^ mice exhibit a less severe renal phenotype during anti-GBM GN

**Fig. 2:**
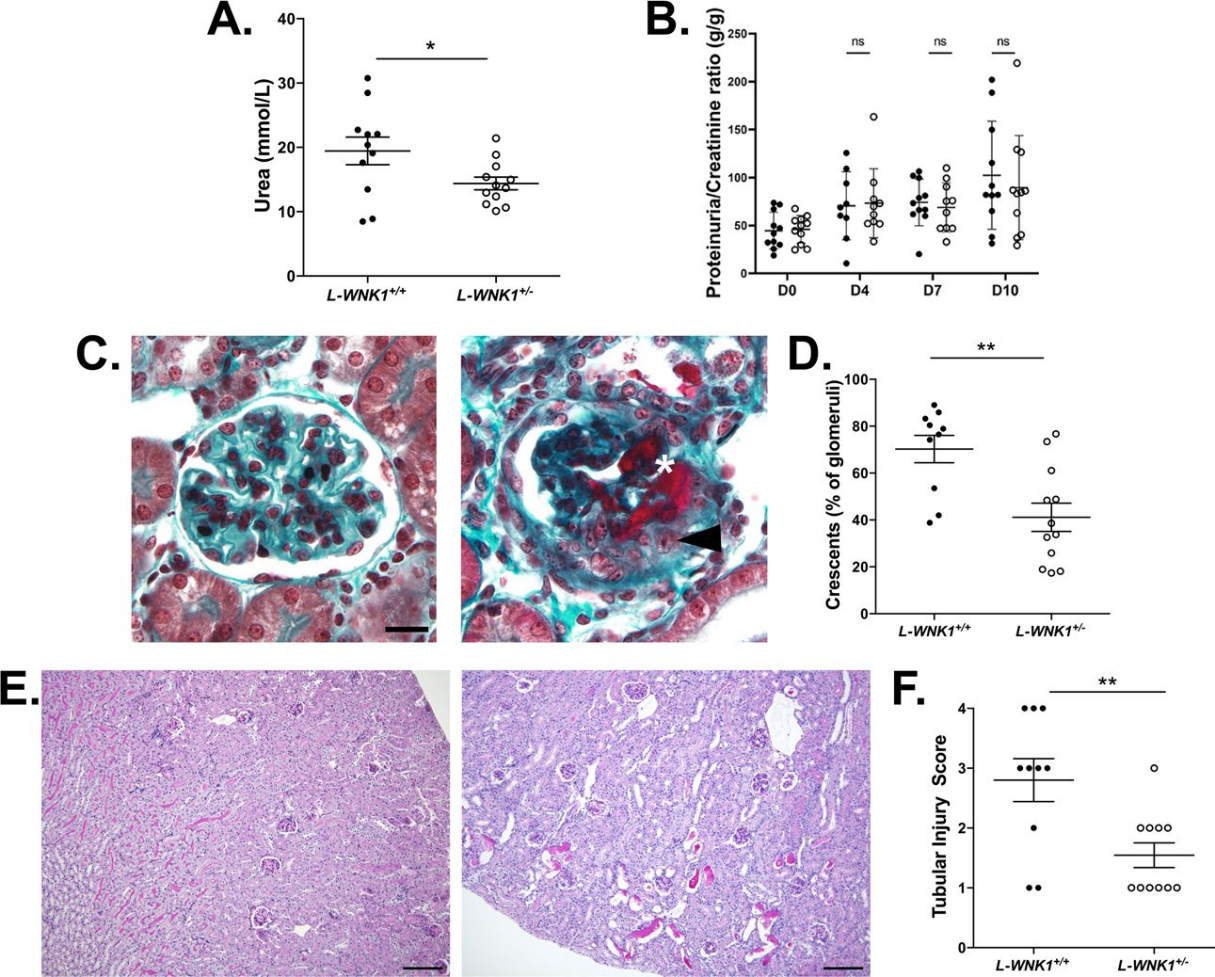
Global L-WNK1 inhibition protects mice from anti-GBM-GN. (**A**) Plasma urea levels in *L-WNK1*^*+/+*^ and *L-WNK1*^*+/-*^ mice on day 10. Data are presented as mean ± SEM (*L-WNK1*^*+/+*^: *n* = 11 - *L-WNK1*^*+/-*^: *n* = 12; Unpaired t-test). (**B**) Urine Protein to urine creatinine ratio of mice at days 0, 4, 7, and 10 post NTS injection. Data are presented as mean ± SEM (*L-WNK1*^*+/+*^: *n* = 11 - *L-WNK1*^*+/-*^: *n* = 12; two-way ANOVA with mixed-effects analysis with a Sidak post-test). (**C**) Representative Masson trichrome staining images of control (left) and NTS-injected (right) mouse kidneys showing glomerular crescents (arrowhead) and fibrin deposits (asterisk). Scale bar: 25 μm. (**D**) Quantification of the percentage of crescentic glomeruli per mouse. Data are presented as mean ± SEM (*L-WNK1*^*+/+*^: *n* = 10 - *L-WNK1*^*+/-*^: *n* = 12; Unpaired t-test). (**E**) Representative Periodic Acid Schiff staining images of control (left) and NTS-injected (right) mouse kidneys showing tubular pseudo-dilations and intra-tubular protein deposits. Scale bar: 200 μm (**F**) Quantification of tubular lesions in both groups. Data are presented as mean ± SEM (*L-WNK1*^*+/+*^: *n* = 10 - *L-WNK1*^*+/-*^: *n* = 11; Unpaired t-test). For all tests, *P < 0.05, **P < 0.01.

Since global deletion of *WNK1* is lethal before embryonic day 13 due to cardiovascular development defects^20^, we used *L-WNK1* heterozygous mice (*L-WNK1*^*+/−*^) to investigate its potential role in RPGN. These mice exhibit no overt phenotypic change on a C57Bl/6J background^21,22^. Upon challenge with the anti-GBM GN model, *L-WNK1*^*+/−*^ mice displayed the expected increase in plasma urea level on day 10. However, this increase was less pronounced compared to L*-WNK1*^*+/+*^ controls (Fig. 2A). The urine protein-to-creatinine ratio (uPCR) rose from day four in both groups in a similar manner (Fig. 2B). Both groups displayed glomerular crescents and acute tubular necrosis lesions (e.g., tubular dilation, cast formation) (Fig. 2C and E). However, *L-WNK1*^*+/−*^ mice had fewer glomerular crescents and tubular lesions compared to controls (Fig. 2D-F). Immunofluorescence analysis revealed no differences in glomerular expression levels of nephrin and WT1 (Fig. S2A-B). WT1 is a podocyte-specific transcription factor, the expression level of which is classically quantified in preclinical models to evaluate the loss of podocytes. Nephrin is the key structural protein of the slit diaphragm. The quantification of its expression is classically used to evaluate the podocyte state during injury.

**Fig. 3:**
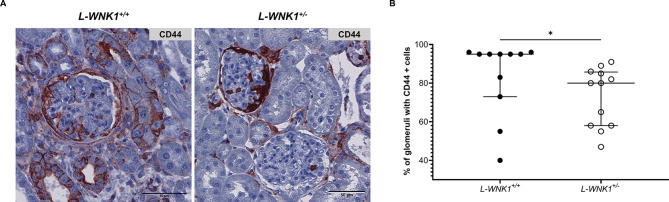
Activation of parietal epithelial cells is reduced when L-WNK1 is inhibited during anti-GBM GN. (**A**) Representative images showing immunochemistry staining of CD44 (brown) in NTS-injected mice. Scale bar: 50 μm. (**B**) Quantification of the percentage of glomeruli with CD44 + cells. Data are presented as mean +/- SEM (Mann-Whitney test - *L-WNK1*^*+/+*^: *n* = 11 - *L-WNK1*^*+/-*^: *n* = 12). *P < 0.05.

The crescent formation is driven mainly by activated parietal epithelial cells proliferating and occluding the glomerular space^14^. Activation of parietal epithelial cells is marked by *de novo* expression of the surface glycoprotein CD44. Consistent with the reduced number of glomerular crescents in *L-WNK1*^*+/−*^ mice, we also observed a lower proportion of glomeruli containing CD44 + cells compared to controls (Fig. 3). These data suggest a protective role for L-WNK1 inhibition in the anti-GBM GN model.

### L-WNK1 Inhibition attenuates pro-inflammatory and pro-fibrotic markers

The anti-GBM GN model is associated with the expression of Monocyte-chemoattractant protein-1 (MCP-1) by macrophages within the glomeruli and tubular compartment^23^. This chemokine promotes monocyte migration and proliferation within inflamed tissues^24^. Following anti-GBM GN induction, MCP-1 expression on day 10 tended to be lower in the kidney of *L-WNK1*^*+/−*^ mice compared to *L-WNK1*^*+/+*^ littermates (Figure S3A).

Anti-GBM GN is also characterized by the upregulation of the expression of pro-fibrotic genes such as *Col3a1* and *Tgfß1*^25,26^. If the transcriptional expression of these genes was elevated on day 10 of anti-GBM GN (Fig. S3B-C), this expression was lower in *L-WNK1*^*+/−*^ kidneys compared to control *L-WNK1*^*+/+*^ animals. Collectively, these data demonstrate that L-WNK1 inhibition reduces the expression of key pro-inflammatory and pro-fibrotic markers in the context of RPGN.

### Mice deficient for WNK1 in podocytes display no renal phenotype in control conditions

The podocytes are one of the primary cells affected during RPGN. Given that *L-WNK1* is highly expressed in the podocytes, we generated a podocyte-specific *WNK1* knockdown (*NPHS2-Cre; WNK1*^*lox/lox*^) mouse by mating mice expressing the Cre recombinase under the dependence of the *NPHS2* promoter with mice carrying a floxed allele of *WNK1* (Fig. S4A-B). 12-week-old *NPHS2-Cre; WNK1*^*lox/lox*^ mice displayed normal renal function and no albuminuria (Fig. S4C-D) and no histopathological change was observed using Masson trichrome stain (Fig. S4E). Immunofluorescence staining for WT1 showed no difference in podocyte number *per* glomerulus at 12 weeks (Fig. S4F-G). Transmission electron microscopy revealed no morphological changes within the capillary loop (Fig. S4H) and the GBM thickness was comparable between *NPHS2-Cre; WNK1*^*lox/lox*^ mice and their littermates (Fig S4I). Thus, WNK1 deletion in podocytes did not appear to affect kidney development and function.

### Podocyte-specific inhibition of WNK1 attenuates anti-GBM GN

**Fig. 4:**
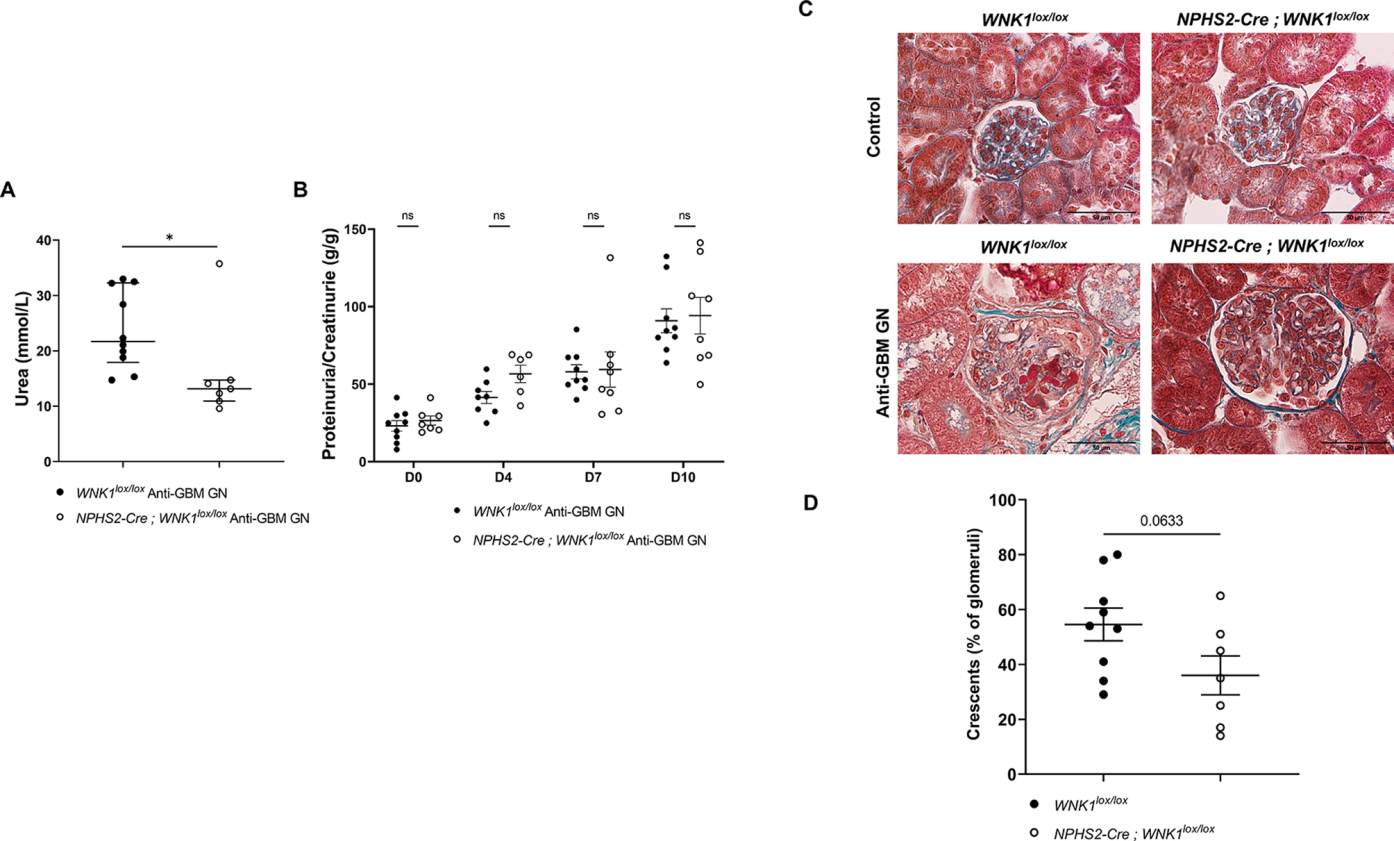
Podocyte-specific inhibition of WNK1 mitigates the phenotype of anti-GBM GN. (**A**) Plasma urea levels in *WNK1*^*lox*/*lox*^ and *NPHS2-Cre; WNK1*^*lox/lox*^ mice at day 10. Data are presented as median+/- interquartile range (Mann Whitney - *WNK1*^*lox/lox*^* n* = 10 - *NPHS2-Cre; WNK1*^*lox/lox*^: *n* = 7). *P < 0.05. (**B**) Urine protein to urine creatinine ratio of mice on days 0, 4, 7, and 10 after NTS injection. Data are presented as+/- SEM (two-way ANOVA with mixed-effects analysis with a Sidak post-test - *WNK1*^*lox/lox*^ *n* = 10 - *NPHS2-Cre; WNK1*^*lox/lox*^: *n* = 8) (**C**) Representative images of Masson trichrome staining in control and NTS injected mice. Glomerular crescents are seen in both anti-GBM GN groups with tubular lesions (tubular dilation, lumen casts). Scale bar: 50 μm. (**D**) Quantification of the percentage of crescentic glomeruli per mouse. Data are presented as mean ± SEM. (Unpaired t-test - *WNK1*^*lox*/*lox*^ *n* = 9 - *NPHS2-Cre; WNK1*^l*ox/lox*^:* n* = 7).

**Fig. 5:**
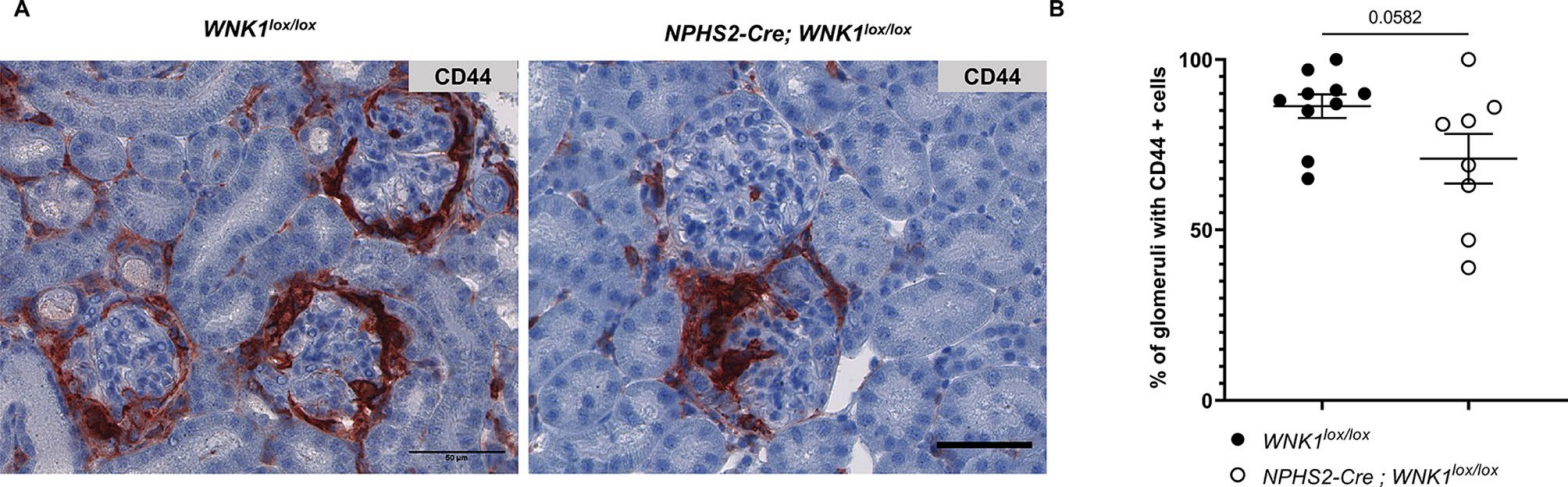
WNK1 inhibition in podocytes limits parietal epithelial cell activation during anti-GBM GN. (**A**) Representative images showing immunochemistry staining of CD44 (brown) in NTS-injected mice. Scale bar: 50 μm. (**B**) Quantification of the percentage of glomeruli with CD44 + cells. Data are presented as mean ± SEM (Unpaired t-test - *WNK1*^*lox/lox*^: *n* = 10 *NPHS2-Cre; WNK1*^*lox/lox*^: *n* = 8).

To investigate the role of podocyte WNK1 in pathological conditions, we induced the anti-GBM GN model in 12-week-old *NPHS2-Cre; WNK1*^*lox/lox*^ mice. Compared to *WNK1*^*lox/lox*^ littermates, *NPHS2-Cre; WNK1*^*lox/lox*^ mice exhibited lower plasma urea levels on day 10 (Fig. 4A). Both groups showed a similar increase in uPCR (Fig. 4B) with no difference in glomerular expression of WT1 and nephrin as assessed by immunostaining (sFig 5 A-B ). *NPHS2-Cre; WNK1*^*lox/lox*^ mice tended to have fewer glomerular crescents (Fig. 4C-D) and fewer CD44+-glomeruli (Fig. 5). In contrast to what is observed in *L-WNK1*^*+/−*^ mice, the podocyte-specific *WNK1* inhibition did not affect *MCP-1* transcription (Figure S6A). This result suggests that the transcription of MCP-1 is modified by L-WNK1 knock-down in another cell type than podocytes in the global inhibition mouse model. However, a significant decrease in Tgf-ß1 transcript level and a trend toward decreased *Col3a1* expression were observed in *NPHS2-Cre; WNK1*^*lox/lox*^ mice (Figure S6B-C). In conclusion, the podocyte-specific knock-down of *L-WNK1* has a protective effect in the context of anti-GBM GN but this effect is less pronounced than the global knock-down. These data suggest that the inhibition of L-WNK1 in another glomerular cell type is involved in its protective effect.

### Inhibition of WNK1 kinase activity reduces podocyte and parietal epithelial cells motility in vitro

To better understand the effects of L-WNK1 inhibition in podocytes, we used the inducible human podocyte cell line AB8/13 ^27^. First, we verified that L-WNK1 was expressed in this cell line using immunofluorescence staining (Fig S7A). Based on the observation that *WNK1* is more expressed in podocytes than *WNK2*, *WNK3* and *WNK4* ^7^, we next decided to use the WNK kinase inhibitor WNK463^28^. As expected, the incubation with WNK463 significantly reduced the phosphorylation of SPAK and OSR1 (Fig. S7B-C and Fig.S9).

A previous study reported that WNK463 disrupted the actin cytoskeleton in podocytes^9^. However, in our study, actin immunostaining did not show any loss of actin stress fiber formation across different cell passages (Fig. S7D).

**Fig. 6:**
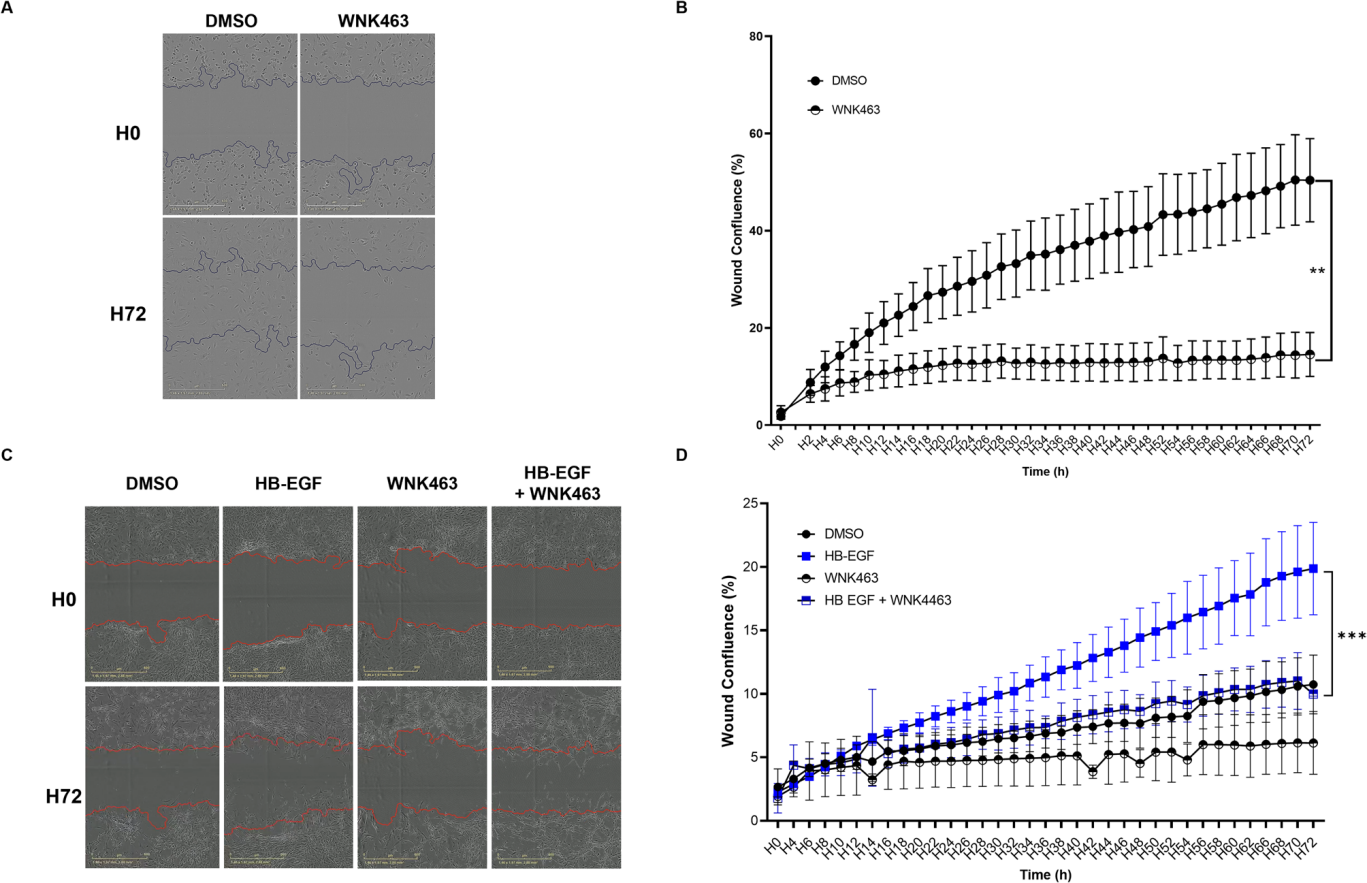
Inhibition of WNK kinase limits podocyte and parietal epithelial cell motility. (**A**) Representative phase-contrast images of human podocytes treated with DMSO or WNK463 (1µM) at H0 and H72. The blue line represents the initial wound. Scale bar: 600 μm. (**B**) Quantification of the wound confluence from t = 0 (0%) to t = 72 h. (**C**) Representative phase-contrast images of murine PECs treated with DMSO, WNK463 (1µM), HB-EGF (10 ng/mL) or HB-EGF with WNK463 at H0 and H72. The red line represents the initial wound. Scale bar: 600 μm. (**D**) Quantification of the wound confluence from t = 0 (0%) to t = 72 h. Data are presented as mean +/- SEM (two-way ANOVA with a Sidak post-test) of three distinct cell passages. **P < 0.01, ***P < 0.001.

Given that L-WNK1 inhibition is associated with a hypomotile phenotype in numerous cell types^18,29^, we performed a wound healing assay with or without WNK463 (Fig. 6A). After 72 h of treatment, WNK kinase inhibition dramatically slowed podocyte migration (Fig. 6B and Video S1).

Since crescent formation involves the proliferation of PECs, we first confirmed that L-WNK1 is expressed in a murine PEC line (Fig. S8). Next, we performed the same wound-healing assay experiments as those conducted in human podocytes in this PEC line. PEC proliferation in culture was induced by adding the growth factor HB-EGF to the medium^30^. However, the addition of WNK463 to the HB-EGF-containing medium significantly reduced PEC confluence at 72 h (Fig. 6C and D and Video S2).

In conclusion, our study suggests that L-WNK1 is a new player in the pathophysiology of RPGN and that its inhibition in podocytes but also possibly in PECs is protective in this pathological context.

## Discussion

We show here that *L-WNK1* is upregulated in glomeruli during RPGN and that global knock-down of L-WNK1 partially protects mice from anti-GBM GN. Additionally, we show that podocyte knockdown of *WNK1* attenuates the effect of anti-GBM GN but the effect of this cell-specific inhibition is less pronounced than the global one. This observation suggests that the expression of L-WNK1 in another cell type is involved in the pathogenesis of anti-GBM GN. Accordingly, we showed that L-WNK1 is critical for the motility of PECs in vitro.

Previous studies reported that L-WNK1 inhibition could be detrimental to murine glomeruli ex vivo and to cultured podocytes^9^. Treatment with the WNK463 inhibitor, as well as with an anti-WNK1 siRNA, resulted in podocyte toxicity by disrupting the actin cytoskeleton. Surprisingly, we did not observe any alteration in actin stress fiber formation in vitro in human podocytes treated with the WNK463 inhibitor. The only obvious difference between the aforementioned study and ours is the podocyte cell line used. Liu and collaborators used a mouse podocyte cell line while we used a human one. However, and perhaps more importantly, we show that knock-down of L-WNK1 in the mouse kidney (both global and podocyte-specific) does not perturb glomerulus development, structure and function. Therefore, if L-WNK1 plays a role in the maintenance of the podocyte actin skeleton in vitro, it does not seem to be crucial in vivo.

The percentage of CD44-positive glomeruli, a surrogate for PEC activation, is reduced in both *L-WNK1*^*+/−*^ and *NPHS2-Cre;WNK1*^*lox/lox*^ mice following anti-GBM GN induction, in the absence of detectable changes in the podocyte markers WT1 and Nephrin. Since PEC activation in RPGN is classically triggered by a modification of podocytes secretome, two hypotheses can be raised. First, functional alterations in podocytes (such as cytokine production) may occur without podocytes dedifferentiation or loss following L-WNK1 inhibition. For example, it is known that the binding of Hb-EGF (Heparin-binding epidermal growth factor-like growth factor) to the EGFR (Epidermal Growth Factor Receptor) in podocytes promotes glomerular injury^31,32^. Jung & Cobb recently showed that depletion of L-WNK1 significantly increased EGFR degradation following ligand stimulation in breast and lung cancer cells^33^. This could explain how L-WNK1 inhibition could modify signalling pathways in podocytes without altering their structure and function. Alternatively, L-WNK1 inhibition could exert direct effects on PECs. This hypothesis is supported by the reduction in PECs activation observed in both mouse models of L-WNK1 inhibition. It is also supported by the inhibition of PECs migration by the WNK inhibitor in vitro. A role for L-WNK1 in the regulation of migration has already been described in T cells in vivo and in *vitro*^18^. This effect is mediated through the phosphorylation of NKCC1 by the SPAK/OSR1 kinases, two known L-WNK1 substrates.

As mentioned above, the inhibition of L-WNK1 in podocytes attenuates the NTS-induced renal disease but to a lesser extent than the global inhibition, strongly suggesting that the kinase could play a role in other cell types contributing to RPGN pathogenesis. If PECs seem a good candidate, one can also speculate that L-WNK1 effect in the context of RPGN could be attributed to immune cells. Indeed, by reducing the migration of T cells, L-WNK1 inhibition could slow down their infiltration of the renal tissue. L-WNK1 inhibition also leads to activation of the NLRP3 inflammasome^34^ and lipopolysaccharide-induced inflammatory responses in macrophages^35^. However, we found that MCP-1 transcript levels were lower in the kidneys of *L-WNK1*^*+/−*^ mice compared to controls in the context of anti-GBM GN. MCP-1 is upregulated in human RPGN, and its urinary levels are correlated with macrophage infiltration in patients^36,37^. The effect on MCP-1 seems independent of L-WNK1 expression in podocytes, as it was observed only in mice with global inhibition of the kinase and not in mice with a podocyte-specific inactivation. A potential link may involve L-WNK1-dependent activation of TGF-β1 expression in tubular cells or mesangial cells^38–41^, which could subsequently induce MCP-1 expression^24^. The role of L-WNK1 in inflammation and fibrogenesis during RPGN warrants further investigation.

In conclusion, we have demonstrated that L-WNK1 is upregulated in RPGN in mice, and we provide evidence for its harmful role in this disease. Inhibiting L-WNK1mitigates the phenotype in an experimental model of RPGN. These findings underscore the importance of further investigating the mechanisms underlying the protective effects of L-WNK1 inhibition in RPGN, especially as pharmacological inhibitors are currently under development.

## Methods

### Human tissues

Acetic acid-formol-alcohol-fixed, paraffin-embedded renal tissue specimens were obtained from the Pathology department of Tenon Hospital, Assistance Publique-Hôpitaux de Paris, Paris, France. All patients used human tissue after informed consent, and the local personal protection committee approved kidney biopsy collection. Patient data used for this research were processed in compliance with French data protection law (“Loi Informatique et Libertés”) and the European General Data Protection Regulation (GDPR, EU 2016/679).

Renal biopsy data were obtained from a certified biobank registered with the French Ministry of Higher Education, Research and Innovation (reference DC-2009-965) and managed by Assistance Publique–Hôpitaux de Paris (AP-HP). All methods were performed in accordance with the relevant guidelines and regulations.

### Animals

All studies were performed according to the European Community Council Directives. The project has been approved by the French Ministry of Research (#22643–2019103010548848). We followed the ARRIVE guidelines. Animals were maintained under standard vivarium conditions, in a pathogen-free animal facility, in ventilated cages with a 12/12 photoperiod, at 22 ± two °C with a 55 ± 10% humidity.

Mice with a heterozygous deletion of *L-WNK1* were generated, described, and kindly provided by Zambrowicz et al. ^21^. They were generated on a mixed genetic background (129/SvEvBrd x C57BL/6J) by gene-trap at the *WNK1* locus. Our mice were obtained after back-crossing *L-WNK1*^*+/−*^ mice with C57BL/6J mice for more than 10 generations. The genotype was determined by PCR using the following primers: L-WNK1-S (5’- AAAATACTCTGTCAGGCTTAAGTGT-3’), L-WNK1-AS (5’- TGAAGCCAGGCATTAAGCACTC-3’) and LTR-reverse (5’-ATAAACCCTCTTGCAGTTGCATC-3’). The generation of *WNK1-*floxed mice has already been described by Hadchouel et al. ^42^. Mice with a specific deletion of *WNK1* in podocytes (*NPHS2-Cre*; *WNK1*^*lox/lox*^) were generated by crossing homozygous *WNK1*^*lox/lox*^ mice^,^ with *NPHS2-Cre*^*+/−*^ mice^43^. These two lines were on a C57BL/6J background. The genotype was determined by PCR using the following primers: Cre-S (5’-CCAGCTCAACATGCTGCACA-3’), Cre-AS (5’-GCCACACCAGACACAGAGAT-3’) for the Cre transgene; WNK1-S (5’-TCTGTTGCCTTTTCTGATGGA-3’) and WNK1-AS (5’-GAAAAGCATACTTCCTCAAACAGAA-3’) for the *WNK1*^*lox*^ allele.

### Anti-GBM GN model

The anti-GBM GN model was induced using decomplemented sheep anti-mouse GBM serum, prepared as previously described, in 2 to 4-months old males^44^. The investigators were not blinded; however, the animal facility staff responsible for animal care were blinded to the experimental conditions. The animals received three retro-orbital intravenous injections of decomplemented nephrotoxic serum (NTS) for three consecutive days. The first injection consisted of a pre-immunization dose of 50 µL of NTS mixed with 50 µL of NaCl 0.9%. The second and third injections consisted of 9 µL/g of NTS. The mice were not randomized since all received the serum, and their only difference was their genotype.

Urine samples were collected prior to the first injection and at four, seven and ten days after the first injection. On day ten, 500 µL of blood were collected via retro-orbital puncture under general anesthesia, using a mixture of ketamine (90 mg/kg) and xylazine (8 mg/kg) diluted in sterile saline to a final volume of 100 µL/10 g body weight, administered intraperitoneally. Mice were subsequently euthanized by cervical dislocation.

### Assessment of renal function and albuminuria

Plasma urea and proteinuria were measured using a Konelab enzymatic-spectrophotometric analyzer (Thermo Fisher Scientific). Creatininuria was assessed via a colorimetric assay based on Jaffe’s reaction with an AU spectrophotometer (SAFAS).

### Histopathology

Mouse kidneys were fixed in AFA (Acetic acid-Formaline-Alcohol) and embedded in paraffin. Section (3-*µ*m thick) were processed for histopathology study, immunohistochemistry, and immunofluorescence. For histopathology, sections were stained with Masson trichrome and Periodic Acid-Schiff stain. All glomeruli of the sections were analyzed. The proportion of crescentic glomeruli (a glomerulus exhibiting more than one layer of cells in Bowman space) was blindly calculated for each mouse. Tubular injury score was quantified by the percentage of damaged tubules (intratubular casts, tubular dilation, tubular atrophy): grade 0, no damage; grade 1, < 25%; grade 2, 25–49%; grade 3, 50–74%; grade 4, ≥ 75%.

For immunohistochemical studies, sections were deparaffinized and incubated for 20 min in a citrate-based target retrieval solution (S2369, Dako, Agilent Technologies) at 100 °C in a pressure cooker (TintoRetriever, BioSB). They were then incubated in peroxidase blocking reagent (S2023, Dako, Agilent Technologies) for 10 min and blocked in TBS-0.1% Tween (TBST) containing 10% bovine serum albumin (BSA, Euromedex). They were then incubated overnight in TBST containing 3% of BSA and the following primary antibodies: rabbit anti-CD44 (1:4000, ab189524, Abcam) and rabbit anti-WNK1 (1:200, HPA059157, Atlas Antibodies). After several washes in TBST, slides were incubated in Histofine reagents containing an anti-rabbit immune-peroxidase polymer (414311 F, Histofine, Nichirei Biosciences) and then counterstained with Mayer’s hematoxylin (TA-125-MH, Richard-Allan Scientific LLC) for 30 s. Images were obtained with optical microscopy (Olympus BX51). The proportion of CD44-positive glomeruli was determinated by examining all glomeruli in each cortical section for every mouse, with assessments by at least two independent experimenters.

### Cell culture

Human AB8/13 podocytes^27^ were cultured in Roswell Park Memorial Institute-1640 (RPMI-1640)-based medium supplemented with 10% fetal bovine serum (FBS; Biosera), and 1% Insulin-Transferrin-Selenium (Invitrogen, Breda). Cells were cultured at 33 °C in a 5% CO_2_ incubator and differentiated at 37 °C for 10–14 days. Following differentiation, podocytes were cultured in the presence or the absence of the WNK inhibitor WNK463 (1µM, MedChemExpress, HY-100626) for 4 h.

Primary mouse PEC line were culture in a RPMI-1640 based medium supplemented with 20% FBS at 37 °C and 5% CO2^45^.

### Wound-healing assay

AB8/13 human podocytes were cultured to confluence in ImageLock 96-well microplates (#4379 Essen BioScience) at 33 °C. After 14 days of differentiation at 37 °C, a precise and reproducible wound was created in each well using the 96-pin IncuCyte^®^ WoundMaker. Following washing steps, podocytes were treated with either DMSO (control) or WNK463 (1 µM) in their culture medium. Migration was monitored by phase-contrast imaging every two hours for three days with an IncuCyte^®^ S3 Live-Cell Analysis System. Wound confluence was calculated using the IncuCyte^®^ S3 software. Three different cell passages were used in the experiment. Similarly, this experiment was also performed with murine PECs at 37 °C, under identical imaging and monitoring conditions using the IncuCyte^®^ system and phase-contrast imaging. However, treatments included either DMSO (control), WNK463 (1 µM), HB-EGF (10 ng/mL - SRP650 Sigma-Aldrich), or WNK463 combined with HB-EGF.

### Statistical analysis

The statistical tests used for the analysis are specified in the figure legends. A difference between groups was considered significant when *p* < 0.05. For quantitative variables, normality was assessed using the Shapiro-Wilk test. If the data followed a Gaussian distribution, parametric tests were applied. In cases of heteroscedasticity, Welch’s test was performed; otherwise, an unpaired t-test was used. Comparisons between multiple groups were conducted using one-way ANOVA. For non-normally distributed data, the Mann-Whitney test was applied. Continuous data comparisons between two groups were performed using two-way ANOVA with Sidak’s post-hoc test. In the presence of heteroscedasticity, a Brown-Forsythe and Welch ANOVA followed by Dunnett’s post-hoc test was conducted. Statistical analyses were performed using GraphPad PRISM software v9 (GraphPad Software).

## Supplementary Information

Below is the link to the electronic supplementary material.

Supplementary Material 1

Supplementary Material 2

Supplementary Material 3

## Data Availability

The data used in this study are available upon reasonable request from the corresponding author.

## References

[1] Veríssimo, F. & Jordan, P. WNK kinases, a novel protein kinase subfamily in multi-cellular organisms. *Oncogene* **20** (39), 5562–5569. 10.1038/sj.onc.1204726 (2001). 11571656 10.1038/sj.onc.1204726

[2] Delaloy, C. et al. Multiple promoters in the WNK1 gene: one controls expression of a kidney-specific kinase-defective isoform. *Mol. Cell. Biol.* **23** (24), 9208–9221. 10.1128/MCB.23.24.9208-9221.2003 (2003). 14645531 10.1128/MCB.23.24.9208-9221.2003 PMC309643

[3] Chen, L., Chou, C. L. & Knepper, M. A. A comprehensive map of mRNAs and their isoforms across all 14 renal tubule segments of mouse. *J Am. Soc. Nephrol. JASN Published Online March*. **4**, ASN2020101406. 10.1681/ASN.2020101406 (2021). 10.1681/ASN.2020101406 PMC8017530 33769951

[4] Thastrup, J. O. et al. SPAK/OSR1 regulate NKCC1 and WNK activity: analysis of WNK isoform interactions and activation by T-loop trans-autophosphorylation. *Biochem. J.* **441** (1), 325–337. 10.1042/BJ20111879 (2012). 22032326 10.1042/BJ20111879 PMC3242505

[5] Gagnon, K. B. & Delpire, E. Molecular physiology of SPAK and OSR1: two Ste20-related protein kinases regulating ion transport. *Physiol. Rev.* **92** (4), 1577–1617. 10.1152/physrev.00009.2012 (2012). 23073627 10.1152/physrev.00009.2012 PMC4519243

[6] Vidal-Petiot, E. et al. A new methodology for quantification of alternatively spliced exons reveals a highly Tissue-Specific expression pattern of WNK1 isoforms. *PLoS ONE*. **7** (5), e37751. 10.1371/journal.pone.0037751 (2012). 22701532 10.1371/journal.pone.0037751 PMC3365125

[7] Park, J. et al. Single-cell transcriptomics of the mouse kidney reveals potential cellular targets of kidney disease. *Science* **360** (6390), 758–763. 10.1126/science.aar2131 (2018). 29622724 10.1126/science.aar2131 PMC6188645

[8] Muto Single cell transcriptional and chromatin accessibility profiling redefine cellular heterogeneity in the adult human kidney. Published online 2020. 10.1101/2020.06.14.151167 10.1038/s41467-021-22368-w PMC8044133 33850129

[9] Liu, Z. et al. Control of podocyte and glomerular capillary wall structure and elasticity by WNK1 kinase. *Front. Cell. Dev. Biol.* **8**, 618898. 10.3389/fcell.2020.618898 (2020). 33604334 10.3389/fcell.2020.618898 PMC7884762

[10] Feng, D. Phosphorylation of key podocyte proteins and the association with proteinuric kidney disease. *Am. J. Physiol-Ren Physiol.* **319** (2), F284–F291. 10.1152/ajprenal.00002.2020 (2020). 10.1152/ajprenal.00002.2020 PMC7528399 32686524

[11] Sethi, S., De Vriese, A. S. & Fervenza, F. C. Acute glomerulonephritis. *Lancet Lond. Engl.* **399** (10335), 1646–1663. 10.1016/S0140-6736(22)00461-5 (2022). 10.1016/S0140-6736(22)00461-5 35461559

[12] Nagata, M. Podocyte injury and its consequences. *Kidney Int.* **89** (6), 1221–1230. 10.1016/j.kint.2016.01.012 (2016). 27165817 10.1016/j.kint.2016.01.012

[13] Ding, M. et al. Loss of the tumor suppressor Vhlh leads to upregulation of Cxcr4 and rapidly progressive glomerulonephritis in mice. *Nat. Med.* **12** (9), 1081–1087. 10.1038/nm1460 (2006). 16906157 10.1038/nm1460

[14] Smeets, B. et al. Tracing the origin of glomerular extracapillary lesions from parietal epithelial cells. *J. Am. Soc. Nephrol. JASN*. **20** (12), 2604–2615. 10.1681/ASN.2009010122 (2009). 19917779 10.1681/ASN.2009010122 PMC2794233

[15] Estrada, C. C. et al. Krüppel-like factor 4 is a negative regulator of STAT3-induced glomerular epithelial cell proliferation. *JCI Insight*. **3** (12), e98214. 10.1172/jci.insight.98214 (2018). 29925693 10.1172/jci.insight.98214 PMC6124441

[16] wei Tu, S., Bugde, A., Luby-Phelps, K. & Cobb, M. H. WNK1 is required for mitosis and abscission. *Proc. Natl. Acad. Sci. U S A*. **108** (4), 1385–1390. 10.1073/pnas.1018567108 (2011). 21220314 10.1073/pnas.1018567108 PMC3029763

[17] Köchl, R. et al. Critical role of WNK1 in MYC-dependent early mouse thymocyte development. *eLife* **9**, e56934. 10.7554/eLife.56934 (2020). 33051000 10.7554/eLife.56934 PMC7591260

[18] Köchl, R. et al. WNK1 kinase balances T cell adhesion versus migration in vivo. *Nat. Immunol.* **17** (9), 1075–1083. 10.1038/ni.3495 (2016). 27400149 10.1038/ni.3495 PMC4994873

[19] Desjardins, P., Couture, C., Germain, L. & Guérin, S. L. Contribution of the WNK1 kinase to corneal wound healing using the tissue-engineered human cornea as an in vitro model. *J. Tissue Eng. Regen Med.* **13** (9), 1595–1608. 10.1002/term.2912 (2019). 31207112 10.1002/term.2912

[20] Xie, J. et al. Endothelial-specific expression of WNK1 kinase is essential for angiogenesis and heart development in mice. *Am. J. Pathol.* **175** (3), 1315–1327. 10.2353/ajpath.2009.090094 (2009). 19644017 10.2353/ajpath.2009.090094 PMC2731149

[21] Zambrowicz, B. P. et al. Wnk1 kinase deficiency lowers blood pressure in mice: a gene-trap screen to identify potential targets for therapeutic intervention. *Proc. Natl. Acad. Sci. U S A*. **100** (24), 14109–14114. 10.1073/pnas.2336103100 (2003). 14610273 10.1073/pnas.2336103100 PMC283554

[22] Bergaya, S. et al. WNK1 regulates vasoconstriction and blood pressure response to α 1-adrenergic stimulation in mice. *Hypertens. Dallas Tex. 1979*. **58** (3), 439–445. 10.1161/HYPERTENSIONAHA.111.172429 (2011). 10.1161/HYPERTENSIONAHA.111.172429 21768522

[23] Lloyd, C. M. et al. RANTES and monocyte chemoattractant protein-1 (MCP-1) play an important role in the inflammatory phase of crescentic nephritis, but only MCP-1 is involved in crescent formation and interstitial fibrosis. *J. Exp. Med.* **185** (7), 1371–1380. 10.1084/jem.185.7.1371 (1997). 9104823 10.1084/jem.185.7.1371 PMC2196251

[24] Haller, H., Bertram, A., Nadrowitz, F. & Menne, J. Monocyte chemoattractant protein-1 and the kidney. *Curr. Opin. Nephrol. Hypertens.* **25** (1), 42–49. 10.1097/MNH.0000000000000186 (2016). 26625862 10.1097/MNH.0000000000000186

[25] Ougaard, M. E. et al. Temporal regulation of glomerular and cortical tubulointerstitial genes involved in the development of nephrotoxic serum nephritis. *Nephron* **140** (3), 218–230. 10.1159/000492294 (2018). 30205387 10.1159/000492294

[26] Meng, X. M., Nikolic-Paterson, D. J. & Lan, H. Y. TGF-β: the master regulator of fibrosis. *Nat. Rev. Nephrol.* **12** (6), 325–338. 10.1038/nrneph.2016.48 (2016). 27108839 10.1038/nrneph.2016.48

[27] Saleem, M. A. et al. A conditionally immortalized human podocyte cell line demonstrating nephrin and Podocin expression. *J. Am. Soc. Nephrol. JASN*. **13** (3), 630–638 (2002). 11856766 10.1681/ASN.V133630

[28] Yamada, K. et al. Small-molecule WNK Inhibition regulates cardiovascular and renal function. *Nat. Chem. Biol.* **12** (11), 896–898. 10.1038/nchembio.2168 (2016). 27595330 10.1038/nchembio.2168

[29] Jaykumar, A. B. et al. WNK1 enhances migration and invasion in breast cancer models. *Mol. Cancer Ther. Published Online July*. **12**10.1158/1535-7163.MCT-21-0174 (2021). 10.1158/1535-7163.MCT-21-0174 PMC9013269 34253593

[30] Lazareth, H. et al. The tetraspanin CD9 controls migration and proliferation of parietal epithelial cells and glomerular disease progression. *Nat. Commun.* **10** (1), 3303. 10.1038/s41467-019-11013-2 (2019). 31341160 10.1038/s41467-019-11013-2 PMC6656772

[31] Bollée, G. et al. Epidermal growth factor receptor promotes glomerular injury and renal failure in rapidly progressive crescentic glomerulonephritis. *Nat. Med.* **17** (10), 1242–1250. 10.1038/nm.2491 (2011). 21946538 10.1038/nm.2491 PMC3198052

[32] Wu, X. et al. Glucocorticoids inhibit EGFR signaling activation in podocytes in Anti-GBM crescentic glomerulonephritis. *Front. Med.* **9**10.3389/fmed.2022.697443 (2022). 10.3389/fmed.2022.697443 PMC8866651 35223886

[33] Jung, J. U. & Cobb, M. H. WNK1 controls endosomal trafficking through TRIM27-dependent regulation of actin assembly. *Proc. Natl. Acad. Sci.* **120** (25), e2300310120. 10.1073/pnas.2300310120 (2023). 37307465 10.1073/pnas.2300310120 PMC10288585

[34] Mayes-Hopfinger, L. et al. Chloride sensing by WNK1 regulates NLRP3 inflammasome activation and pyroptosis. *Nat. Commun.* **12** (1), 4546. 10.1038/s41467-021-24784-4 (2021). 34315884 10.1038/s41467-021-24784-4 PMC8316491

[35] Arai, Y. et al. WNK1–TAK1 signaling suppresses lipopolysaccharide-induced cytokine production and classical activation in macrophages. *Biochem. Biophys. Res. Commun.* **533** (4), 1290–1297. 10.1016/j.bbrc.2020.10.007 (2020). 33046244 10.1016/j.bbrc.2020.10.007

[36] Wada, T. et al. MIP-1alpha and MCP-1 contribute to crescents and interstitial lesions in human crescentic glomerulonephritis. *Kidney Int.* **56** (3), 995–1003. 10.1046/j.1523-1755.1999.00646.x (1999). 10469367 10.1046/j.1523-1755.1999.00646.x

[37] Tam, F. W. K. et al. Urinary monocyte chemoattractant protein-1 (MCP-1) is a marker of active renal vasculitis. *Nephrol. Dial Transpl. Off Publ Eur. Dial Transpl. Assoc. - Eur. Ren. Assoc.* **19** (11), 2761–2768. 10.1093/ndt/gfh487 (2004). 10.1093/ndt/gfh487 15353578

[38] Li, Y. et al. OSR1 phosphorylates the Smad2/3 linker region and induces TGF-β1 autocrine to promote EMT and metastasis in breast cancer. *Oncogene* **40** (1), 68–84. 10.1038/s41388-020-01499-2 (2021). 33051597 10.1038/s41388-020-01499-2

[39] Jaykumar, A. B. et al. WNK1 collaborates with TGF-β in endothelial cell junction turnover and angiogenesis. *Proc. Natl. Acad. Sci. U S A*. **119** (30), e2203743119. 10.1073/pnas.2203743119 (2022). 35867836 10.1073/pnas.2203743119 PMC9335306

[40] Lee, B. H., Chen, W., Stippec, S. & Cobb, M. H. Biological Cross-talk between WNK1 and the transforming growth factor β-Smad signaling Pathway*. *J. Biol. Chem.* **282** (25), 17985–17996. 10.1074/jbc.M702664200 (2007). 17392271 10.1074/jbc.M702664200

[41] Qi, W. et al. TGF-beta1 induces IL-8 and MCP-1 through a connective tissue growth factor-independent pathway. *Am. J. Physiol. Ren. Physiol.* **290** (3), F703–709. 10.1152/ajprenal.00254.2005 (2006). 10.1152/ajprenal.00254.2005 16204411

[42] Hadchouel, J. et al. Decreased ENaC expression compensates the increased NCC activity following inactivation of the kidney-specific isoform of WNK1 and prevents hypertension. *Proc. Natl. Acad. Sci.* **107** (42), 18109–18114. 10.1073/pnas.1006128107 (2010). 20921400 10.1073/pnas.1006128107 PMC2964238

[43] Moeller, M. J., Sanden, S. K., Soofi, A., Wiggins, R. C. & Holzman, L. B. Podocyte-specific expression of Cre recombinase in Transgenic mice. *Genes N Y N 2000*. **35** (1), 39–42. 10.1002/gene.10164 (2003). 10.1002/gene.10164 12481297

[44] Mesnard, L. et al. Invariant natural killer T cells and TGF-beta attenuate anti-GBM glomerulonephritis. *J. Am. Soc. Nephrol. JASN*. **20** (6), 1282–1292. 10.1681/ASN.2008040433 (2009). 19470687 10.1681/ASN.2008040433 PMC2689902

[45] Kabgani, N. et al. Primary cultures of glomerular parietal epithelial cells or podocytes with proven origin. *PloS One*. **7** (4), e34907. 10.1371/journal.pone.0034907 (2012). 22529955 10.1371/journal.pone.0034907 PMC3329559

