# Supplementary material for: Knock-down of the long isoform of the WNK1 kinase mitigates the anti-glomerular basement membrane glomerulonephritis in mice
Source: Sci Rep. 2026 Feb 5;16:7335. doi: 10.1038/s41598-026-36715-8 (PMC12923593; doi:10.1038/s41598-026-36715-8)
Supplement: Supplementary file 3 — Supplementary Material 3 [file 41598_2026_36715_MOESM3_ESM.docx]

**Supplementary Files**

**Supplementary Methods.**

*Nephrin and WT1 immunofluorescence*

Paraffin-embedded mice kidneys were deparaffinized and incubated for 20 minutes in the target retrieval solution pH6 (S2369 Dako, Agilent Technologies) at 100°C in a pressure cooker and blocked in TBS-0.1% Tween containing 10% bovine serum albumin (BSA, Euromedex). Sections were incubated overnight in a humidified chamber at four °C with the following primary and secondary antibodies: guinea pig anti-nephrin (1:100, GP-N2, progren) and rabbit anti-WT1 (1:100, ab89901, Abcam).

*Transmission electron microscopy*

Kidneys were fixed in 2.5% glutaraldehyde in 0.1 mol/L cacodylate buffer (pH 7.4) at four °C for 24 hours and then post-fixed in 1% OsO4 for one hour, dehydrated using graded alcohol series, and embedded in epoxy resin. Semi-thin sections (0.5mm) were stained by toluidine blue, and 60-nm ultrathin sections were contrasted with uranylless (Delta Microscopies) and lead citrate. Images were obtained with a JEOL 1010 electron microscope (JEOL, Ltd) with a MegaView III camera (Olympus Soft Imaging Systems GmbH). The mean GBM thickness (nm) was measured using a method adapted from^1^. Each mouse analyzed at least two glomeruli and ten capillary loops per glomerulus. GBM thickness was measured on 10 points per capillary loop by an examiner blinded to the experimental conditions. GBM thickness measurements were then averaged for each mouse

*Isolation of glomeruli*

Glomeruli were isolated by two-step sieving of renal cortices. Kidneys were decapsulated, cut into small pieces, and digested for 3 minutes at 37°C in collagenase I (2 mg/ml, 17100-017, Gibco, ThermoFisher Scientific, Waltham, MA, USA) in RPMI 1640 (Gibco, ThermoFisher Scientific, Waltham, MA, USA). The collagenase I was then inactivated by the addition of RPMI 1640 containing 10% of Fetal Calf Serum (FCS, BioSera, Nuaille, France). The digested tissue was then passed through a 70-μm cell strainer (BD falcon) on a 50-mL tube. The filter was flushed with PBS+0.5% BSA and then discarded. Next, the 50-ml tube containing tubules and glomeruli was shaken several times and passed through a 40-μm cell strainer (BD falcon). Glomeruli adherent to the 40-μm cell strainer were taken from the cell strainer with PBS+0.5% BSA injected under pressure, then washed in PBS. Isolated glomeruli were then frozen at −80°C.

*Total RNA extraction, reverse transcription, and quantitative RT-PCR*

Total kidney extracts and dry pellets of glomeruli were lysed and homogenized in TRIzol reagent (MRC). Phase separation was made using BAN (BN 191, MRC). RNA was precipitated with isopropanol and washed with 70% ethanol. After air drying, the pellet was resuspended in nuclease-free water. Following this step, a DNase (EN0521, ThermoFisher Scientific, Waltham) treatment was performed for 30 minutes at 37°C. Reverse transcription (K1642, ThermoFisher Scientific) was then made using 200 ng of RNA per sample. Differential gene expression was quantified by real-time PCR performed in a 96-well format using the light cycler 480 SYBR Green I Master (Roche Life Science), the Bio-Rad Connect Real-Time detection system apparatus, and the CFX Maestro 2.2 software (Version 5.2.008.0222). All the samples were assayed in duplicate, and the average value of the duplicate was used for quantification. Relative gene expression levels were calculated after normalization with housekeeping genes. Primers for mice were used. Calculations were made using the Pfäffl method^2^. The sequences of primers used in our studies are listed in Supplemental **Table S2**.

*CRISPR-Cas9 WNK1 knockout in immortalized human podocytes*

An immortalized human podocyte cell line carrying a loss-of-function mutation in the *WNK1* gene was generated using the CRISPR-Cas9 system. The sgRNA sequence (GCACTCTGCGGGACAGCCGC) targeting exon 1 of *WNK1* previously described in the HEK293T cell line was used^3^. Immortalized human podocytes kindly provided by Moin Saleem were transfected at 33 °C with the pSpCas9(BB)-2A-GFP (PX458) plasmid containing the sgRNA^4^. This plasmid enables expression of both the sgRNA and Cas9 nuclease. Two days after transfection, single-cell clones were isolated using a cell sorter (Hana Single Cell Dispenser, Namocell). Among the resulting clones, one carrying a one-nucleotide deletion in exon 1, resulting in a frameshift and a premature stop codon at position 125, was selected.

*Western blot analysis*

Protein lysates from AB8/13 podocytes were prepared with RIPA extraction buffer containing phosphatase and protease inhibitors (Roche). Total protein concentration was measured using the BCA protein assay kit (23225/23227, ThermoFisher Scientific). Ten µg of proteins were loaded onto polyacrylamide electrophoresis gels for separation and transferred onto nitrocellulose membranes. The membranes were blocked with milk and probed with different antibodies: rabbit anti-WNK1 (1:1000, HPA059157, Atlas Antibodies), rabbit anti-pSPAK/pOSR1 (Ser373/Ser325) (1:1000, 07-2273, Millipore), and rabbit anti-GAPDH (1:20000, #G9545, Sigma). Membranes were then probed with horseradish peroxidase-conjugated secondary antibodies (NA9340, donkey anti-rabbit, Amersham), and bands were visualized by enhanced chemiluminescence (Clarity Western ECL substrate; Bio-Rad, 170–5061). A ChemiDoc MP imaging system (12003154, Biorad) was used to reveal bands, and densitometric analysis was used for quantification.

*Immunofluorescence staining*

Differentiated AB8/13 human podocytes and primary mouse PEC line were fixed with 1% paraformaldehyde, permeabilized with PBS containing 0.5% Triton, and blocked with PBS containing 3% BSA. The following primary antibodies were used: rabbit anti-WNK1 (1:200, HPA059157, Atlas Antibodies) and FITC-conjugated phalloidin (1:400, ActiStain, Cytoskeleton Inc). The secondary antibody used was donkey anti-rabbit IgG AF 594-conjugated (1:750, A-21207, Life Technologies). Nuclei were counterstained with DAPI (1:4000, 62248, ThermoFisher Scientific) for 3 minutes. All images were processed and analyzed using ImageJ software (version 1.53c).

**Supplementary reference**

**S**1. Marquez B, Zouvani I, Karagrigoriou A, Anastasiades E, Pierides A, Kyriacou K. A simplified method for measuring the thickness of glomerular basement membranes. *Ultrastruct Pathol*. 2003;27(6):409-416.

S2. Pfaffl MW. A new mathematical model for relative quantification in real-time RT–PCR. *Nucleic Acids Res*. 2001;29(9):e45.

S3. Roy A, Goodman JH, Begum G, et al. Generation of WNK1 knockout cell lines by CRISPR/Cas-mediated genome editing. *Am J Physiol Renal Physiol*. 2015;308(4):F366-376. doi:10.1152/ajprenal.00612.2014

S4. Saleem MA, O’Hare MJ, Reiser J, et al. A conditionally immortalized human podocyte cell line demonstrating nephrin and podocin expression. *J Am Soc Nephrol JASN*. 2002;13(3):630-638.

**Supplementary Table S1. Human Kidney Biopsies: diagnosis and patients characteristics**

|  | **Control: Acute Tubular Necrosis** | **Anti-GBM GN** | **ANCA vasculitis** |
| --- | --- | --- | --- |
| *Age* | 43 | 46 | 19 |
| *Sex* | F | F | M |
| *Serum Creatinine (mg/dL)* | 7.3 | 6.4 | 5.8 |
| *Urine Protein to Creatinine Ratio (g/g)* | 0.6 | 2 | 1.4 |

**Supplementary Table S2. Primer Sequences for Real-Time PCR**

| **Transcript** | **Forward Primer** | **Reverse Primer** |
| --- | --- | --- |
| *Gusb* | CTCTGGTGGCCTTACCTGAT | CAGTTGTTGTCACCTTCACCTC |
| *HPRT* | CCTCCTCAGACCGCTTTTT | AACCTGGTTCATCATCGCTAA |
| *UBQ* | AGCCCAGTGTTACCACCAAG | ACCCAAGAACAAGCACAAGG |
| *WNK1 ex 3S-3/5AS* | TGGTCCTACTGGCTCAGTCA | GGCTGGCTTCACTCCCTATC |
| *MCP-1* | GTTGGCTCAGCCAGATGCA | AGCCTACTCATTGGGATCATCTTG |
| *TGF-β1* | TGGAGCAACATGTGGAACTC | GTCAGCAGCCGGTTACCA |
| *COL3α1* | TCCCCTGGAATCTGTGAATC | TGAGTCGAATTGGGGAGAAT |


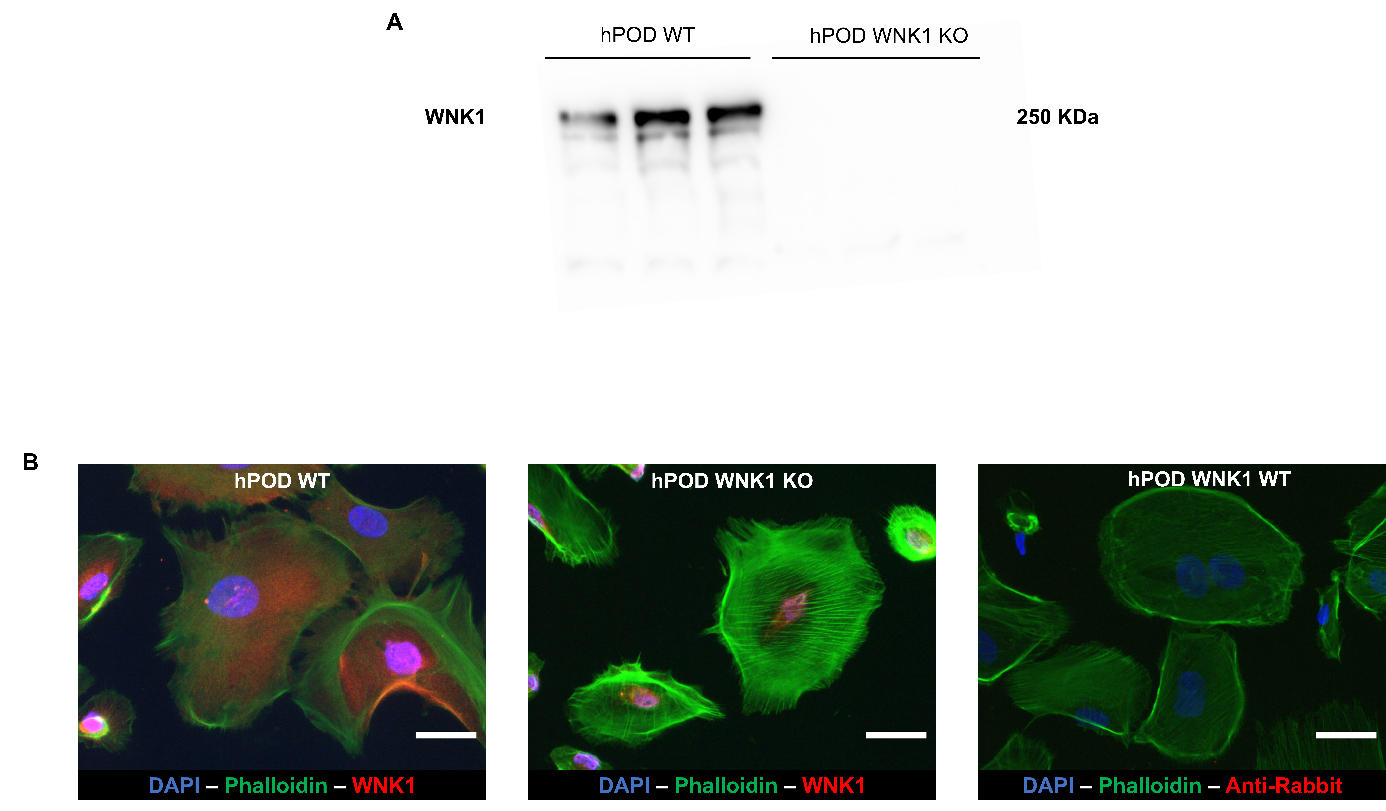


**Supplementary Figure S1. Validation of L-WNK1 antibody specificity in human WNK1^-/-^ podocytes.** A. Analysis of L-WNK1 protein expression in control and L-WNK1-deficient human podocytes across three different cell passages. B. Immunofluorescence staining of the actin cytoskeleton (phalloidin, green), WNK1 (red), and nuclear counterstaining with DAPI in control podocytes (left panel), L-WNK1-deficient podocytes (middle panel), and rabbit isotype control (right panel). Representative images from at least three independent passages. Scale bar: 50 μm


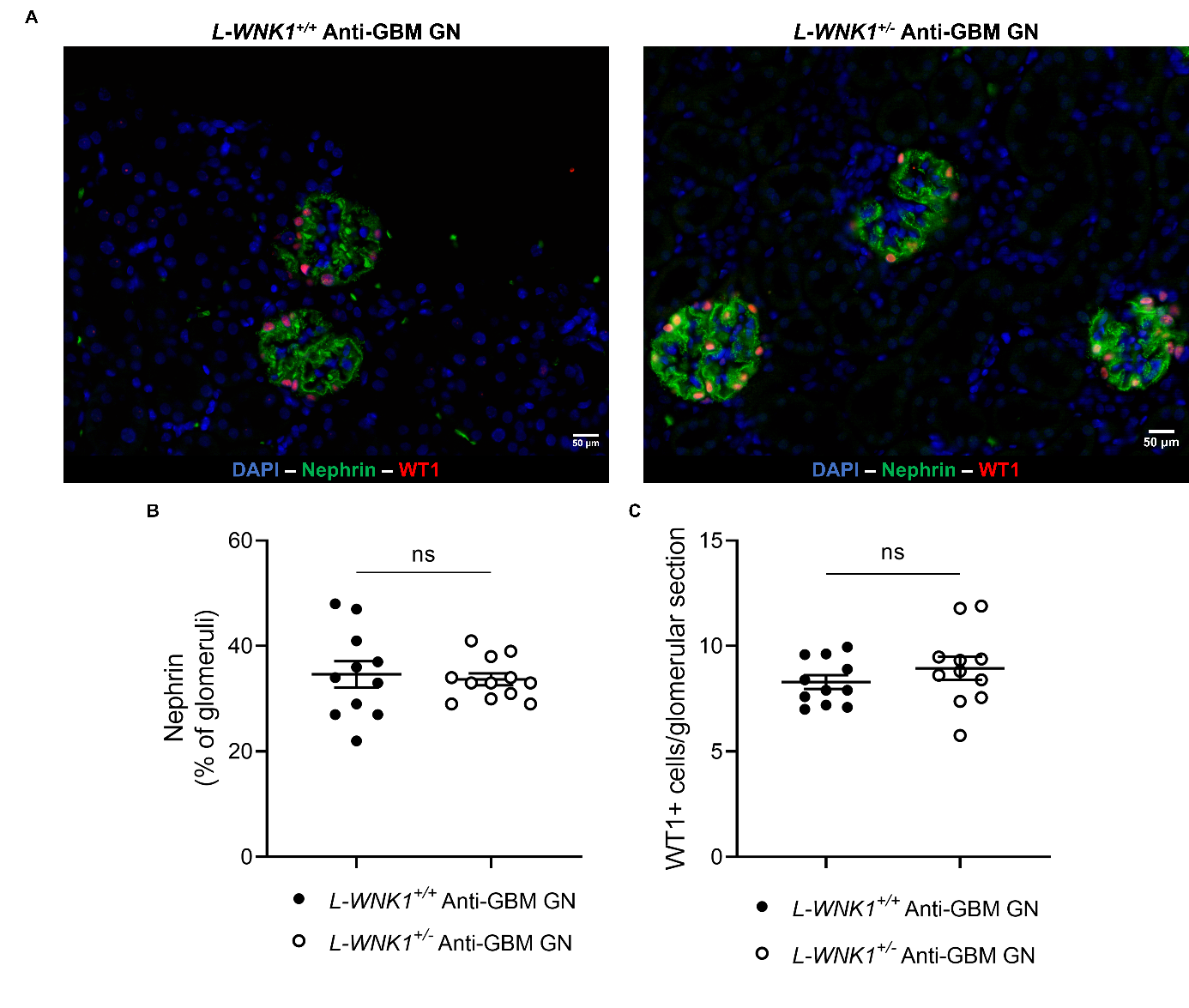


**Supplementary Figure S2. Global inhibition of *WNK1* does not modify nephrin and WT1 expression during anti-GBM GN.** A. Representative images showing immunofluorescent stainings of WT1 (red), Nephrin (green), and DAPI (blue) in 12-weeks-old *L-WNK1^+/+^* and L-*WNK1^+/-^* mice affected by anti-GBM GN. Scale bar: 50 µm. B. Quantification of the surface of glomeruli stained by nephrin.(Welch’s t-test - *L-WNK1^+/+^*: n = 11 - L-*WNK1^+/-^*: n = 12). C. Quantification of WT1+ cells per glomerular section. (Unpaired t-test - *L-WNK1^+/+^*: n = 11 - L-*WNK1^+/-^*: n = 12).


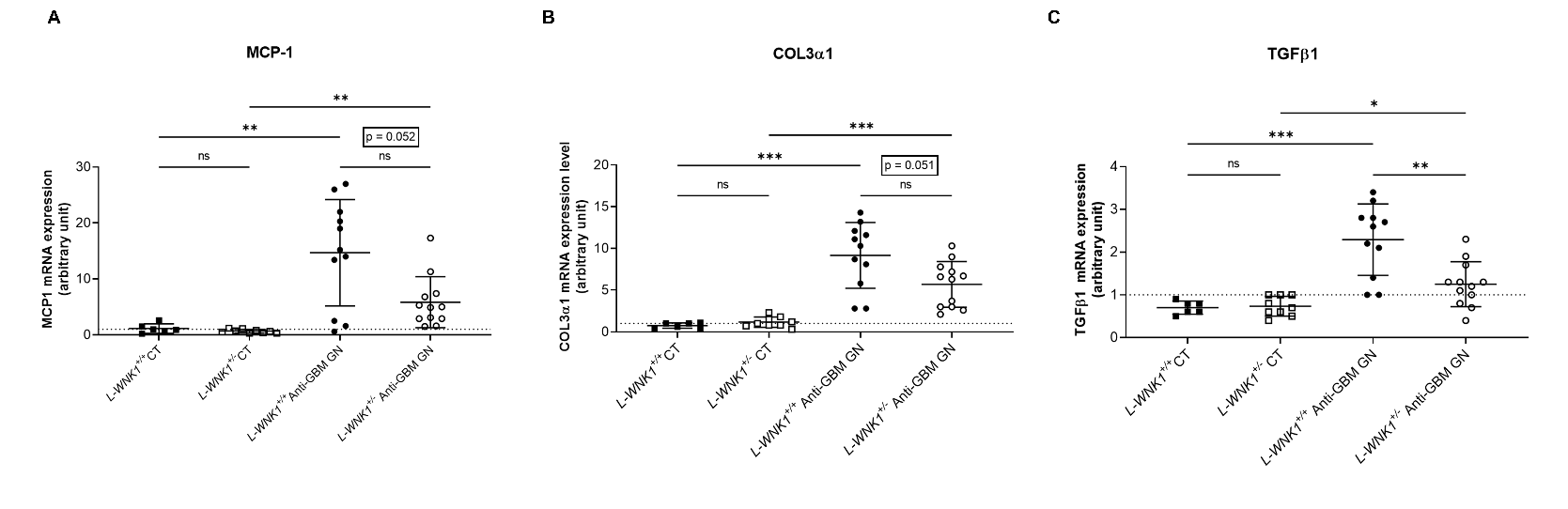


**Supplementary Figure S3. L-WNK1 inhibition decreases pro-inflammatory and pro-fibrosis markers during anti-GBM GN.** A. mRNA expression of MCP-1 in total kidney extract. B. mRNA expression of COL3α1 in total kidney extract. C. mRNA expression of TGFβ1 in total kidney extract. The data represents the mean +/- standard deviation (Brown-Forsythe and Welch ANOVA with a Dunnet post-test). L-WNK1^+/+^ control (n = 6), L-WNK1^+/-^ control (n = 9), L-WNK1^+/+^ anti-GBM- GN(n=11) and L-WNK1^+/-^ (n=12) anti-GBM- GN mice. *P<0.05, **P<0.01, ***P<0.001.


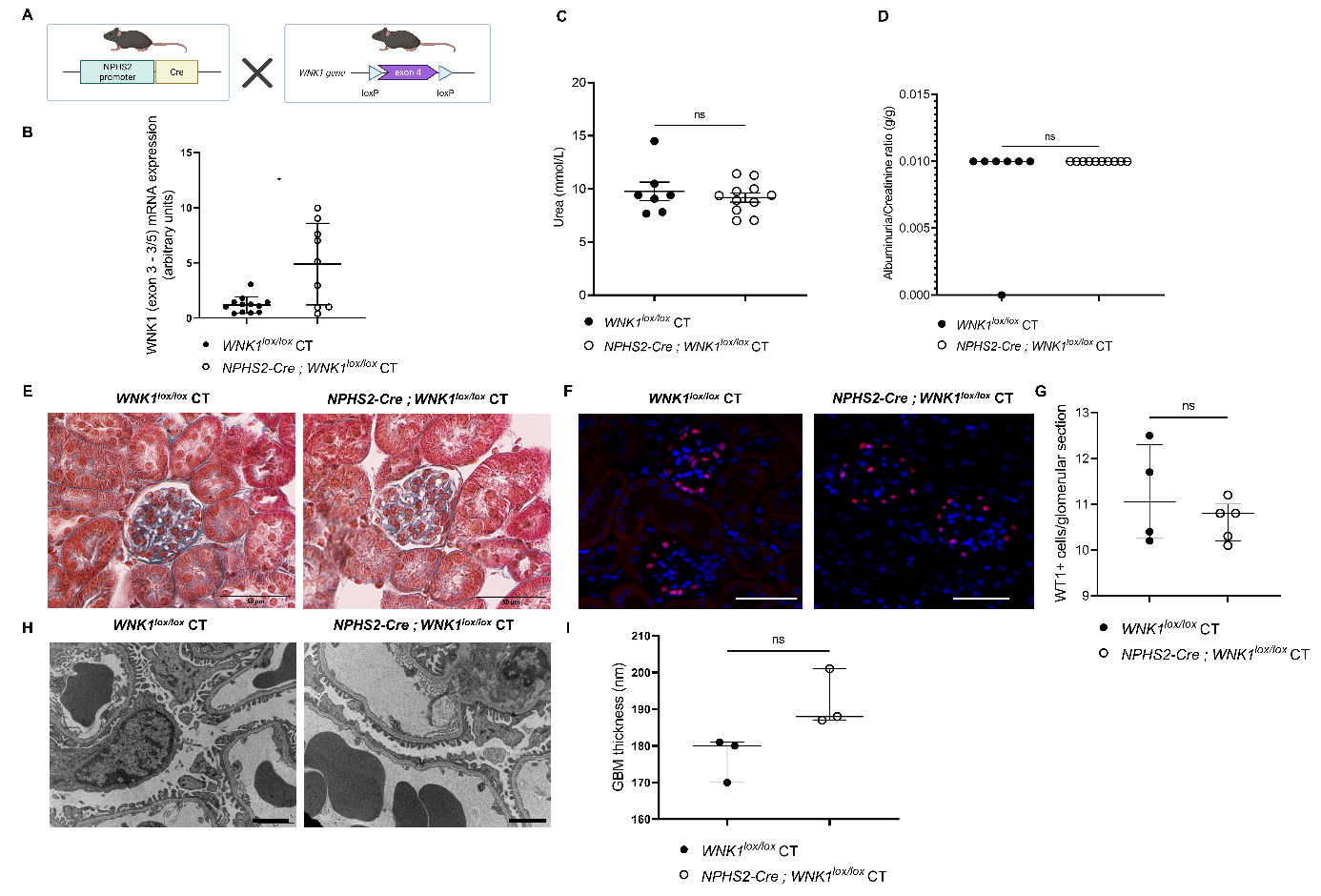


**Supplementary Figure S4. Podocyte knockdown of *WNK1* does not induce glomerular damage.** A. Generation of *NPHS2-Cre ; WNK1^lox/lox^* mice were obtained by mating mice expressing a heterozygous Cre recombinase under the dependence of the *NPHS2* promoter with mice expressing the exon 4 of *WNK1* gene flanked by two loxP sequences. Deletion of exon 4 leads to a premature codon stop. Figure created with Biorender.com. B. mRNA expression of *WNK1* transcript deleted from exon 4 in isolated glomeruli. *P<0.05 (Welch’s t-test - *WNK1^lox/lox^*: n = 12 - *NPHS2-Cre ; WNK1^lox/lox^*: n = 9). C. Plasmatic urea levels in 12-weeks-old *WNK1^lox/lox^* and *NPHS2-Cre ; WNK1^lox/lox^* mice at basal state. The data represents the mean +/- standard error of the mean (Unpaired t-test - *WNK1^lox/lox^*: n = 7 - *NPHS2-Cre ; WNK1^lox/lox^*: n = 11). D. Urine albumin to urine creatinine ratio of *WNK1^lox/lox^* and *NPHS2-Cre ; WNK1^lox/lox^* mice at basal state. (Mann-Whitney test - *WNK1^lox/lox^*: n = 7 - *NPHS2-Cre ; WNK1^lox/lox^*: n = 10). E. Representative images of Masson trichrome staining of 12-weeks-old *WNK1^lox/lox^* and *NPHS2-Cre ; WNK1^lox/lox^* mice at basal state. (*WNK1^lox/lox^*: n = 7 - *NPHS2-Cre ; WNK1^lox/lox^*: n = 11) Scale bar: 50 µm. F. Representative images showing immunofluorescent stainings of WT1 (red) and DAPI (blue) at baseline in 12-weeks-old *WNK1^lox/lox^* and *NPHS2-Cre ; WNK1^lox/lox^* mice. Scale bar: 50 µm. G. Quantification of WT1+ cells per glomerular section in 12-weeks-old *WNK1^lox/lox^* and *NPHS2-Cre ; WNK1^lox/lox^* mice. (Mann-Whitney test - *WNK1^lox/lox^*: n = 4 - *NPHS2-Cre ; WNK1^lox/lox^*: n = 5). H. Ultrastructural analysis of glomerular basement membrane (GBM) by transmission electron microscopy (TEM). Representative electron micrographs of glomerular sections from 12-weeks-old *WNK1^lox/lox^* and *NPHS2-Cre ; WNK1^lox/lox^* mice. Scale bar: 0.5µm. I. Quantification of GBM thickness of *WNK1^lox/lox^* and *NPHS2-Cre ; WNK1^lox/lox^* mice. (Mann-Whitney test - *WNK1^lox/lox^*: n = 3 - *NPHS2-Cre ; WNK1^lox/lox^*: n = 3)


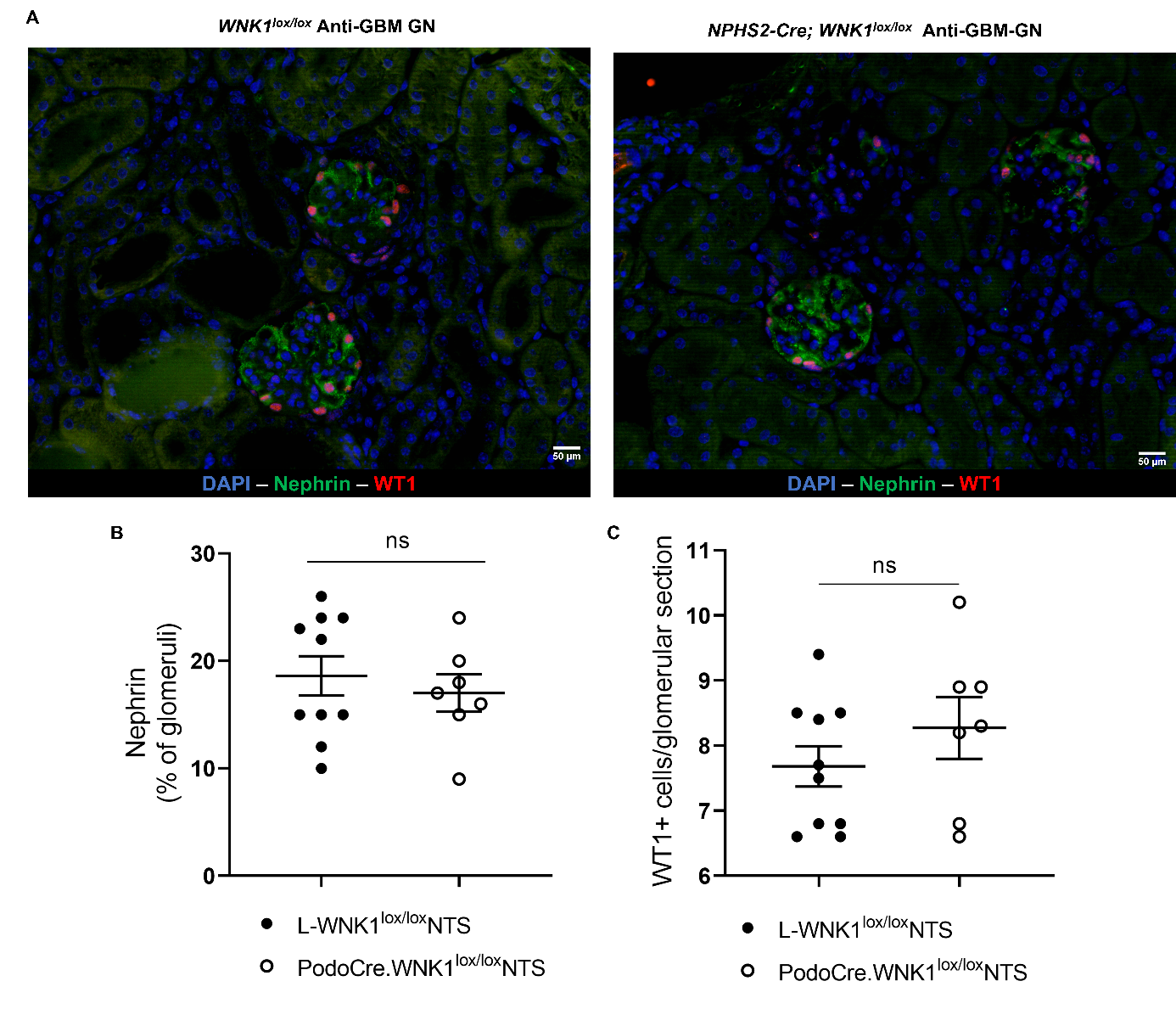


**Supplementary Figure S5. Podocyte knockdown of *WNK1* does not modify nephrin and WT1 expression during anti-GBM GN.** A. Representative images showing immunofluorescent stainings of WT1 (red), Nephrin (green), and DAPI (blue) in 12-weeks-old *WNK1^lox/lox^* and *NPHS2-Cre ; WNK1^lox/lox^* mice affected by anti-GBM GN. Scale bar: 50 µm. B. Quantification of the surface of glomeruli stained by nephrin.(Unpaired t-test - *WNK1^lox/lox^*: n = 10 - *NPHS2-Cre ; WNK1^lox/lox^*: n = 7). C. Quantification of WT1+ cells per glomerular section. (Unpaired t-test - *WNK1^lox/lox^*: n = 10 - *NPHS2-Cre ; WNK1^lox/lox^*: n = 7).


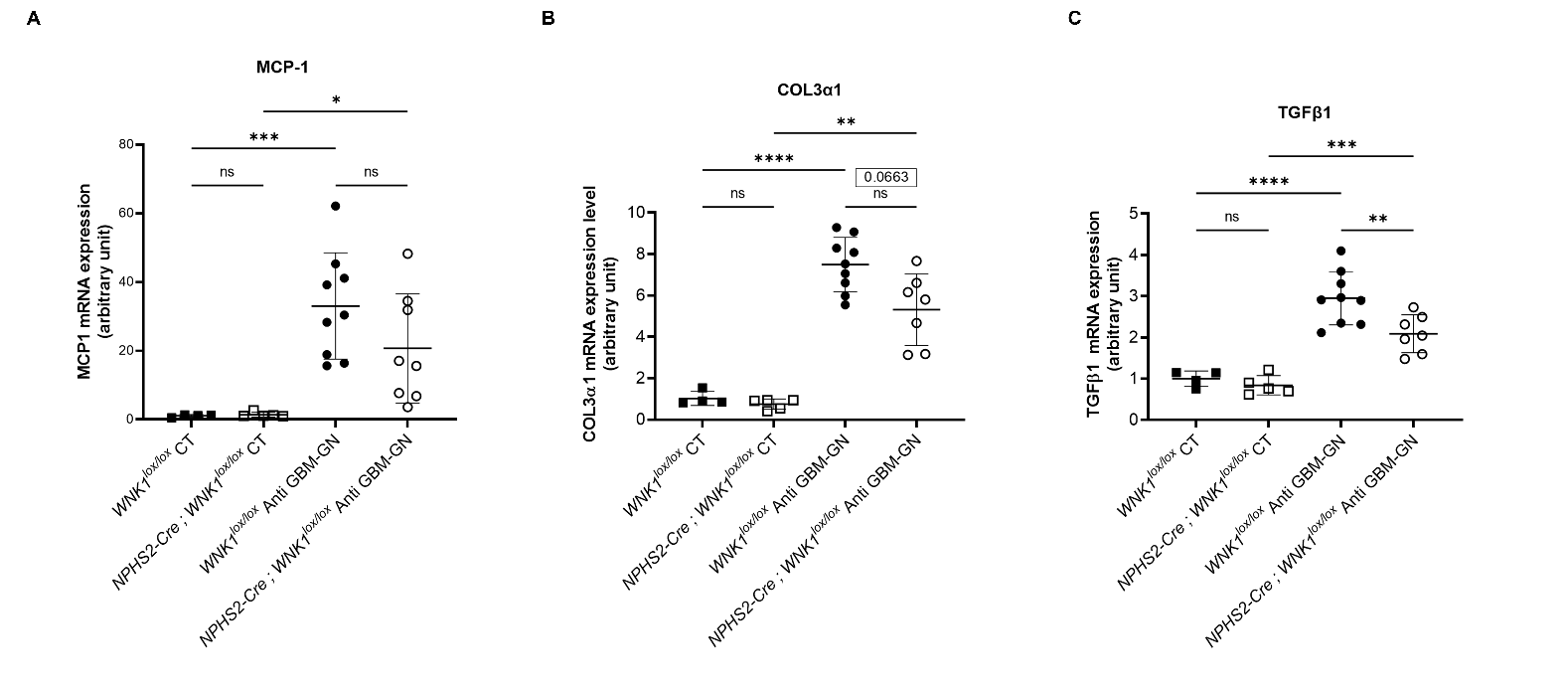


**Supplementary Figure S6. Effect of WNK1 podocyte deletion on pro-inflammatory and pro-fibrosis markers during anti-GBM GN*.*** *A.* mRNA expression of *MCP-1* in total kidney extract. B. mRNA expression of *COL3α1* in total kidney extract. C*.* mRNA expression of *TGFβ1* in total kidney extract*.* The data represents the mean +/- standard deviation (Ordinary one-way ANOVA with a Sidak post-test). *WNK1^lox/lox^* control (n = 4)*,* NPHS2-Cre ; WNK1^lox/lox^ control (n = 5)*, WNK1^lox/lox^* anti-GBM- GN(n=9) and *NPHS2-Cre ; WNK1^lox/lox^* (n=8) anti-GBM- GN mice*.* **P<0.05, **P<0.01, ***P<0.001, ****P<0.0001*.

**
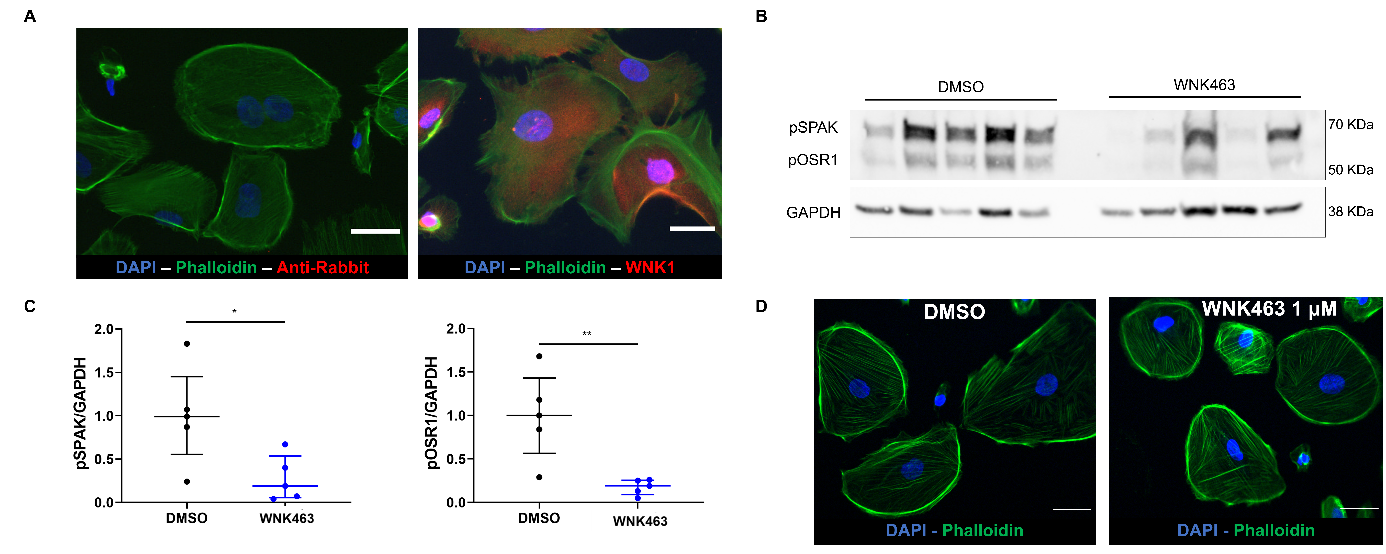
**

**Supplementary Figure S7. WNK1 and its mediators are expressed in human podocytes.** A. Representative images immunofluorescent staining for WNK1 or its negative control (red), phalloidin (green), and DAPI (blue) on human podocytes (AB8/13 cells). WNK1 expression is cytoplasmic. Scale bar: 50 µm. B. Western blot analysis of phospho-SPAK (Ser373) and phospho-OSR1(Ser325) of human podocytes treated or not with WNK463 (1µM). GAPDH serves as a housekeeping protein. C. Quantification of phosphoSPAK/GAPDH and phosphoOSR1/GAPDH ratio. Data are presented as mean +/- standard deviation (Mann Whitney test). N= 5 for all groups). *P<0.05, **P<0.01. D. Representative images immunofluorescent staining for phalloidin (green) and DAPI (blue) on human podocytes (AB8/13 cells) treated with DMSO or WNK463 1µM. WNK1 expression is cytoplasmic. Scale bar: 50 µm.


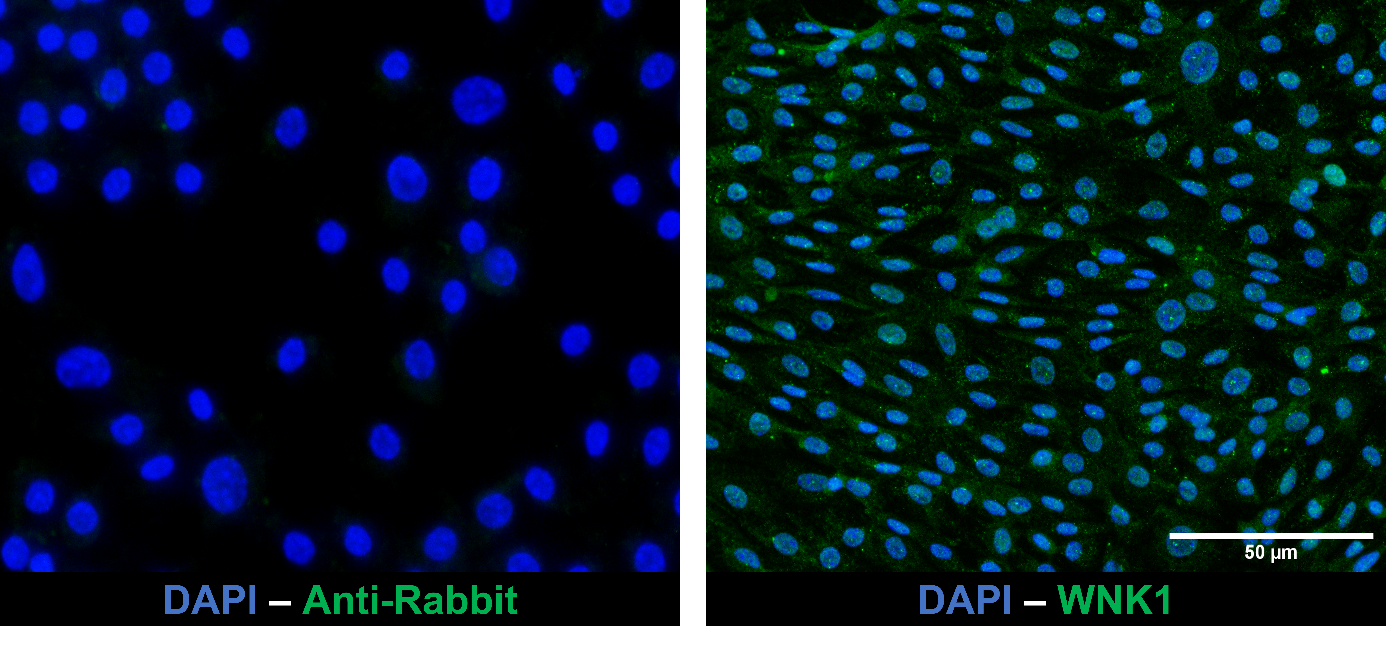


**Supplementary Figure S8. WNK1 is expressed in cultured mouse PEC.** Representative images immunofluorescent staining for WNK1 or its negative control (green) and DAPI (blue) on mouse PEC. Scale bar: 50 µm


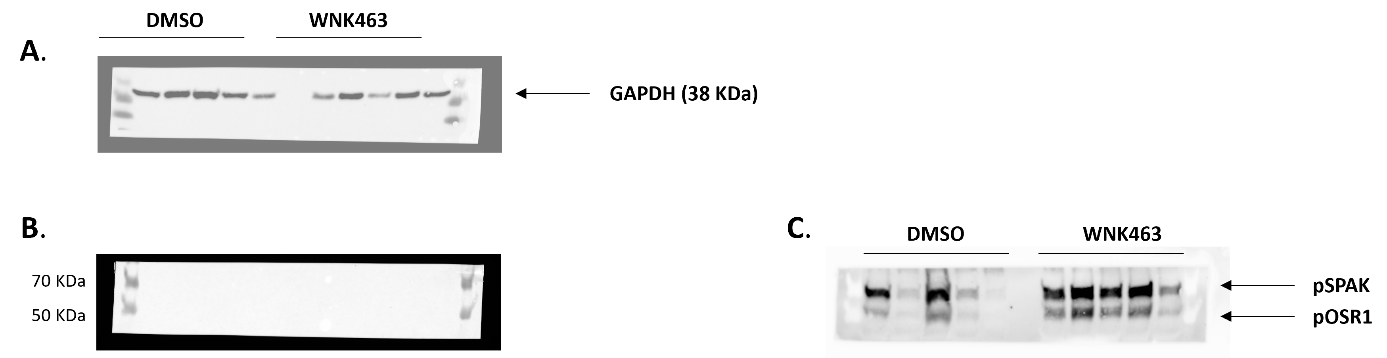


**Supplementary Figure S9. Uncropped gel for Figure S7.** Western blot analysis of the expression of pSPAK and OSR1 in podocytes AB8/13 cell line treated with DMSO or WNK463 1µM. A. GAPDH with Ponceau stain combined. B. Ponceau staining alone. C. pSPAK and pOSR1.


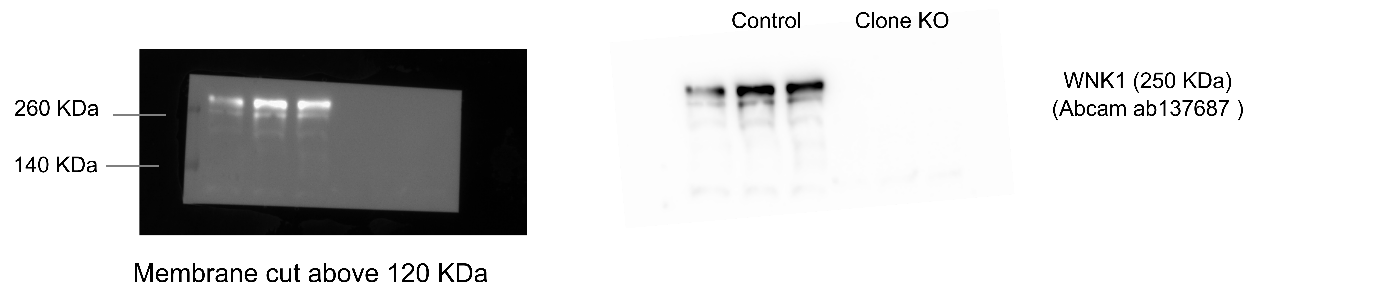


**Supplementary Figure S10.** Uncropped gel for Figure S1. Western blot analysis of the expression of WNK1 in podocytes AB8/13 cell line (WT and WNK1^-/-^).

**Supplementary Videos S1.** Representative time-lapse of wound healing assay in human podocytes with DMSO (A) or WNK463 (B). Images are taken every 2 hours for 72 hours. The blue line represents the initial wound. Scale bar: 600 µm.

**Supplementary Videos S2.** Representative time-lapse of wound healing assay in mouse PEC with DMSO (A), HB-EGF (B), WNK463 (C) or WNK463 with HB-EGF (D). Images are taken every 2 hours for 72 hours. The red line represents the initial wound. Scale bar: 600 µm.
